# Antibiotic use and immune-related adverse events in patients treated with immune checkpoint inhibitors: analysis of the FAERS database

**DOI:** 10.3389/fimmu.2026.1733373

**Published:** 2026-05-05

**Authors:** Jia Yu, Qinxiao Li, Shangpu Zou, Yiyin Rong, Yuting Zhang, Chengshui Chen

**Affiliations:** 1 Zhejiang Province Engineering Research Center for Endoscope Instruments and Technology Development, Department of Pulmonary and Critical Care Medicine, Quzhou People’s Hospital, The Quzhou Affiliated Hospital of Wenzhou Medical University, Quzhou, China; 2 Department of Dermatology, Second Affiliated Hospital of Xi’an Jiaotong University, Xi’an, China; 3 Department of Respiratory and Critical Care Medicine, The First Affiliated Hospital of Wenzhou Medical University, Wenzhou, China; 4 Key Laboratory of Interventional Pulmonology of Zhejiang Province, Department of Pulmonary and Critical Care Medicine, The First Affiliated Hospital of Wenzhou Medical University, Wenzhou, China

**Keywords:** adverse event reporting system (FAERS), antibiotics, cancer, immune checkpoint inhibitors, immune-related adverse events

## Abstract

**Background:**

The use of antibiotics may influence the efficacy and toxicity of immune checkpoint inhibitors (ICIs) by altering the gut microbiota. However, current evidence on the link between antibiotic use and immune-related adverse events (irAEs) is limited. This study aims to evaluate whether antibiotics increase the risk of irAEs in ICI-treated patients and to examine their relationship to the timing of irAEs onset.

**Methods:**

We analyzed data from the FAERS database from 2014 to the fourth quarter of 2024. Using multivariable logistic regression and descriptive statistical analyses, we evaluated the association between antibiotic co-reporting and irAE reporting frequency and the timing across different antibiotic categories and ICIs regimens.

**Results:**

Our study included 155,157 patients treated with ICIs, of whom 9,518 (6.1%) received antibiotic therapy. Patients who used antibiotics had a significantly higher reported frequency risk of irAEs (OR = 1.17; 95%CI: 1.12–1.23; FDR<0.001) compared to those who did not. The strongest associations were observed in patients receiving fluoroquinolones, sulfonamides, penicillin, macrolides, cephalosporins, and monobactams. Co-reporting was associated with a higher reported frequency of irAEs in patients receiving PD-L1 inhibitors (OR = 1.51; 95% CI: 1.39–1.65; FDR<0.001). In exploratory descriptive analysis restricted to patients who reported irAEs, the median time to first reported irAE was shorter in the antibiotic co-reporting group than in the non-co-reporting group (31 days (IQR: 9–105) vs. 42 days (IQR: 14–122), Wilcoxon rank-sum test P < 0.001). Stratified analysis by ICI type showed that this pattern was most evident in patients receiving PD-1 inhibitors.

**Conclusions:**

Analysis of the FAERS database suggests that antibiotic co-reporting during ICIs therapy is associated with a higher reported frequency of irAEs and a shorter median time to first reported irAE among patients who experienced irAEs. These findings are subject to the inherent limitations of the FAERS database, including the inability to determine the temporal sequence of antibiotic and ICI exposure, unmeasured confounding, reporting artifacts, and the unsuitability of spontaneous reporting data for formal time-to-event analysis. Prospective cohort studies with detailed medication timing, clinical phenotyping, and microbiome profiling are needed to validate these signals.

## 1 Introduction

Cancer cells can evade the immune system through immune checkpoint pathways (1). Immune checkpoint inhibitors (ICIs) fight tumors by binding to proteins like programmed cell death ligand 1 (PD-L1), programmed cell death protein 1 (PD-1), and cytotoxic T lymphocyte-associated protein 4 (CTLA-4), which are present on cell surfaces. This action helps activate T cells (2–5). ICIs are now widely adopted in clinical practice and have greatly improved survival rates in many cancers (6). However, excessive activation of the immune system by ICIs can lead to autoimmune toxicity, and this cross-organ systemic immune response activation may trigger immune-related adverse events (irAEs) (7–9). IrAEs can occur at any time during ICIs therapy, with severe irAEs necessitating discontinuation of immunotherapy and potentially posing life-threatening risks (10). Identifying risk factors for irAE is crucial for balancing the benefits and risks of immunotherapy. These irAEs impact patients’ quality of life and may also be deadly, so modifiable risk factors must be identified for better clinical management.

Previous studies show that antibiotics lower the efficacy of immunotherapy (11–15). This may occur because antibiotics disrupt gut microbiota diversity, leading to dysbiosis. Gut microbiotas play a critical role in maintaining immune homeostasis and shaping the efficacy of ICIs (16). However, evidence linking antibiotic exposure to irAEs remains limited. Studies have identified increased rates of immune-mediated colitis associated with antibiotic use (17, 18); though these investigations are predominantly single-center, small-sample studies with significant population heterogeneity. Furthermore, systematic analysis of the temporal relationship between antibiotic exposure and irAEs onset, as well as potential differential effects of different antibiotic classes on irAEs risk, remains an unaddressed gap.

To address this evidence gap, we conducted a large-scale pharmacovigilance analysis using the FDA Adverse Event Reporting System (FAERS) database (19). This study aimed to evaluate the association between antibiotic co-reporting and the reported frequency and timing of irAEs in patients receiving ICIs therapy. We further explored whether these associations differed across antibiotic classes and ICIs regimens. Given the inherent limitations of spontaneous reporting databases, our findings are intended as hypothesis-generating safety signals that may inform future prospective investigations into antibiotic stewardship during immunotherapy.

## 2 Methods

### 2.1 Study design and reporting guidelines

We used a cross-sectional design to analyze FAERS reports for associations between antibiotic use and irAEs during ICIs therapy. This study followed the STROBE checklist for cross-sectional studies. The completed checklist is in **Supplementary Table 1** (20).

### 2.2 Data repository

Our data comes from FAERS, an open-access pharmacovigilance platform managed by the U.S. Food and Drug Administration (FDA). The database contains adverse events from drugs after they are released. This dataset is publicly available at https://fis.fda.gov/extensions/FPD-FAQ/FPD-FAQ.html#_Toc514144622.

### 2.3 Data acquisition and filtering

In this study, adverse event reports related to ICIs were retrieved from the FAERS database, covering the period from 2014 to December 30, 2024. The dataset included records of the FDA approval date for each ICI through the end of 2024. To ensure data quality, we employed the FDA-recommended deduplication algorithm to remove duplicate reports based on case identifiers, report numbers, and patient demographic information. When multiple reports existed for the same adverse event, only the most recent report was retained. The FAERS database categorizes drugs into four roles in adverse events: primary suspect (PS), secondary suspect (SS), concomitant (C), or interacting (I). We included all patients receiving ICIs labeled as PS, excluding reports lacking data on age, gender, drug usage, adverse reaction type, or indication.

**Figure 1:**
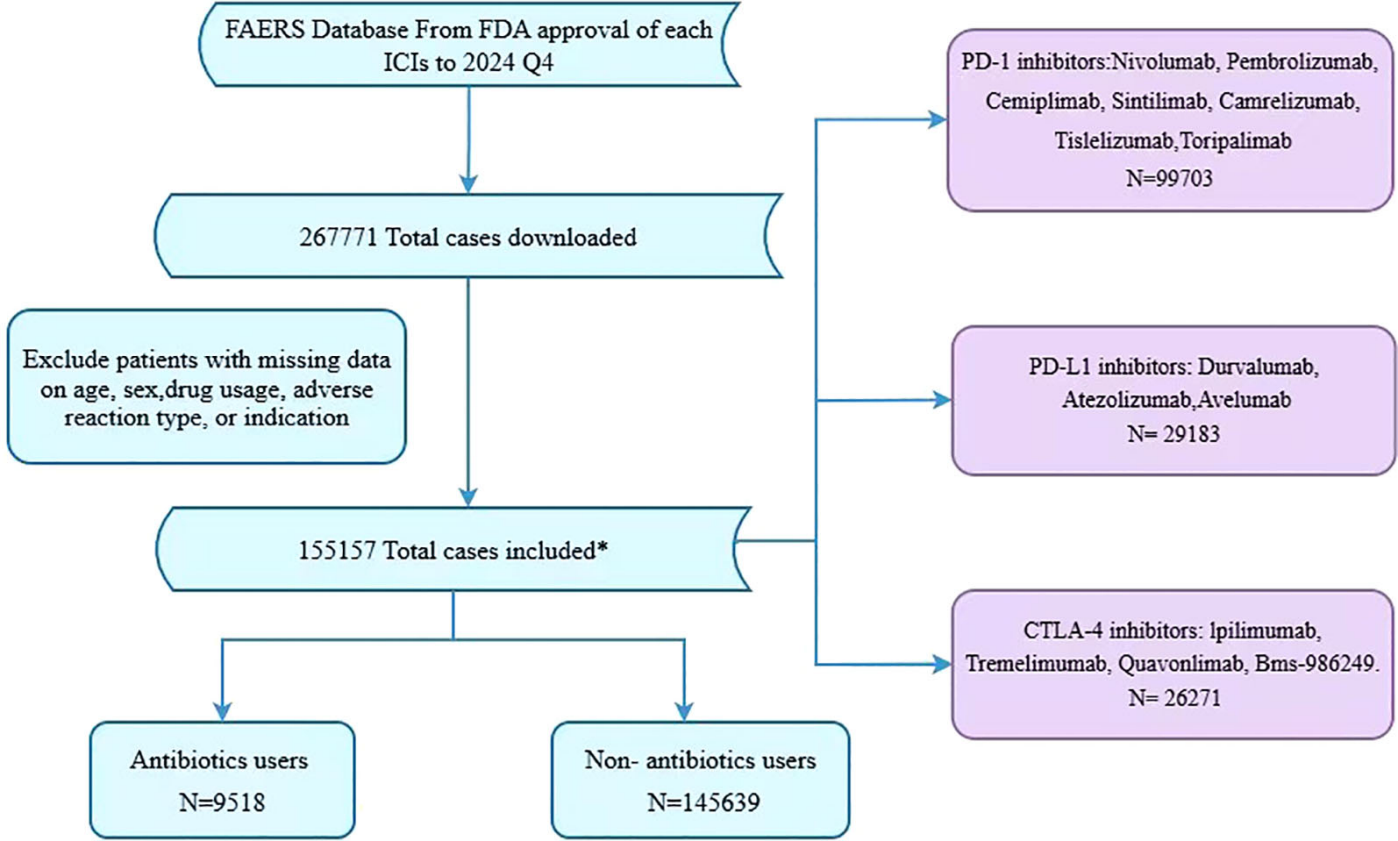
Flow chart showing the analysis process of the study. All treatment regimens were included: monotherapy (n=32,729, 21%), multiple ICIs combination (n=90,290, 58%), and chemotherapy plus ICIs (n=32,138, 21%). For patients receiving multiple ICIs, classification was based on the PS drug in the FAERS database. FDA, Food and Drug Administration; ICI, Immune checkpoint inhibitor.

The ICIs of interest were PD-1 inhibitors (such as nivolumab, Camrelizumab, Cemiplimab, Sintilimab, pembrolizumab, Toripalimab, and Tislelizumab), PD-L1 inhibitors (such as durvalumab, Avelumab, and atezolizumab), and CTLA-4 inhibitors (Tremelimumab, Quavonlimab, and ipilimumab). Treatment regimens were categorized according to composition into monotherapy, multi-immunotherapy, and chemo-immunotherapy combinations. All three treatment regimens were included in the analysis. Monotherapy was defined as the use of a single ICIs agent (PD-1, PD-L1, or CTLA-4 inhibitor). multi-immunotherapy referred to the combination of two or three different ICIs. Chemo-immunotherapy was defined as the combination of ICIs and cytotoxic chemotherapy. If a patient concurrently used multiple ICIs, classification into ICIs type groups was based on the ICIs labeled as PS. This classification method complies with the guidelines for pharmacovigilance studies in the FAERS. Due to inherent limitations in the FAERS database structure, it is not possible to precisely determine the temporal relationship between antibiotic use and the initiation of ICIs therapy, nor to confirm whether antibiotic exposure preceded, coincided with, or followed the onset of irAEs. Therefore, patients who had any systemic antibiotic recorded as a concomitant medication in their FAERS report were classified into the antibiotic co-reporting group. This classification reflects co-reporting rather than a confirmed temporal exposure sequence, and the possibility of reverse causation (i.e., antibiotics prescribed to treat infections arising from irAE management) cannot be excluded. Twelve major classes of antibiotics were analyzed: penicillins, cephalosporins, aminoglycosides, macrolides, fluoroquinolones, sulfonamides, monobactams, lincosamides, tetracyclines, carbapenems, glycopeptides, and others (such as metronidazole and polymyxin B). The specific drug names and search terms used to identify each antibiotic class in the FAERS database are provided in **Supplementary Table 2**. According to published peer-reviewed guidelines (18, 21), patients are considered to have experienced an irAE if they develop any adverse event listed in **Supplementary Table 3**. Adverse events are coded according to the System Organ Class (SOC) of the Medical Dictionary for Regulatory Activities (MedDRA, Version 27.1) (22). A schematic flowchart of the study process is presented in **Figure 1**.

### 2.4 Statistical analysis

To evaluate the association between antibiotic use and both the risk and timing of irAEs onset, multivariable logistic regression models were applied, adjusting for potential confounders including age, sex, ICI type, treatment regimen, and reporter country. To control the risk of false discovery due to multiple testing, the Benjamini–Hochberg procedure was implemented using the p.adjust function in the R “stats” package. Two-sided tests were performed, and an FDR-adjusted p value (FDR p) < 0.05 was considered statistically significant. To characterize the temporal pattern of irAE occurrence, we calculated the median and interquartile range (IQR) of the time from ICIs initiation to first reported irAE for both the antibiotic and non-antibiotic groups. The Wilcoxon rank-sum test (Mann–Whitney U test) was used to compare the onset time distributions between groups. Box plots and histograms were used to visualize these distributions. Stratified analyses by ICI type (PD-1, PD-L1, CTLA-4) were performed with the Kruskal–Wallis test. These descriptive approaches were chosen because FAERS is a spontaneous reporting system that lacks verified treatment start dates, defined follow-up windows, and formal censoring mechanisms.

## 3 Results

### 3.1 Initial patient characteristics

We downloaded safety reports for 155,157 patients receiving ICIs therapy from FAERS, among whom 9,518 (6.1%) reported baseline use of antibiotics, as shown in **Table 1**. Analysis of this subgroup revealed a higher proportion of males among antibiotic users: 5,678 (60%) males versus 3,840 (40%) females. The mean age for both antibiotic users and non-users was 65 years. Age stratification showed that 46% of antibiotic users were aged 18–64 years, 39% were aged 65–75 years, and 14% were over 75 years. In contrast, among non-users, 45% were aged 18–64 years, 37% in the 65–75 age group, and 16% in the over-75 age group. The most common treatment strategy across the entire population was the use of PD-1 inhibitors, followed by PD-L1 inhibitors and CTLA-4 inhibitors. The countries with the highest numbers of adverse event reports were the United States and Japan, indicating either higher reporting rates or potentially greater use of these therapies in these nations.

**Table 1:** Baseline feature.

Characteristic	Overall (N = 155157)	Without antibiotic, (N = 145639)	With antibiotic, (N = 9518)	p-value
Age (SD^1^)	65 ± 14	65 ± 14	65 ± 13	0.4
Age_group				<0.001
0-17	1,985 (1.3%)	1,922 (1.3%)	63 (0.7%)	
18-64	70,444 (45%)	66,078 (45%)	4,366 (46%)	
65-75	57,843 (37%)	54,122 (37%)	3,721 (39%)	
>=75	24,868 (16%)	23,500 (16%)	1,368 (14%)	
Sex				<0.001
Female, n (%)	60,032 (39%)	56,192 (39%)	3,840 (40%)	
Male, n (%)	95,125 (61%)	89,447 (61%)	5,678 (60%)	
ICIs type				<0.001
CTLA-4	26,271 (17%)	24,427 (17%)	1,844 (19%)	
PD-1	99,703 (64%)	95,031 (65%)	4,672 (49%)	
PD-L1	29,183 (19%)	26,181 (18%)	3,002 (32%)	
Treatment regimen				<0.001
Monotherapy	32,729 (21%)	29,724 (20%)	3,005 (32%)	
Multi-ICI	90,290 (58%)	86,454 (59%)	3,836 (40%)	
Chemo-ICI	32,138 (21%)	29,461 (20%)	2,677 (28%)	
Reporter country				<0.001
United States	42,633 (27%)	39,971 (27%)	2,662 (28%)	
Japan	34,432 (22%)	33,172 (23%)	1,260 (13%)	
France	17,976 (12%)	17,105 (12%)	871 (9.2%)	
Germany	8,822 (5.7%)	8,027 (5.5%)	795 (8.4%)	
China	6,683 (4.3%)	6,409 (4.4%)	274 (2.9%)	
Canada	5,548 (3.6%)	5,045 (3.5%)	503 (5.3%)	
Italy	4,301 (2.8%)	4,106 (2.8%)	195 (2.0%)	
United Kingdom	3,743 (2.4%)	3,106 (2.1%)	637 (6.7%)	
Spain	3,460 (2.2%)	2,997 (2.1%)	463 (4.9%)	
Other Country	27498 (18%)	25632 (17.6%)	1866 (19.6%)	
irAEs				<0.001
No	114,418 (74%)	107,552 (74%)	6,866 (72%)	
Yes	40,739 (26%)	38,087 (26%)	2,652 (28%)	

^1^ Standard Deviation (SD) for continuous; n (%) for categorical, irAEs, immune-related Adverse Events.

### 3.2 Association of antibiotic use with irAEs in patients receiving ICIs

**Figure 2:**
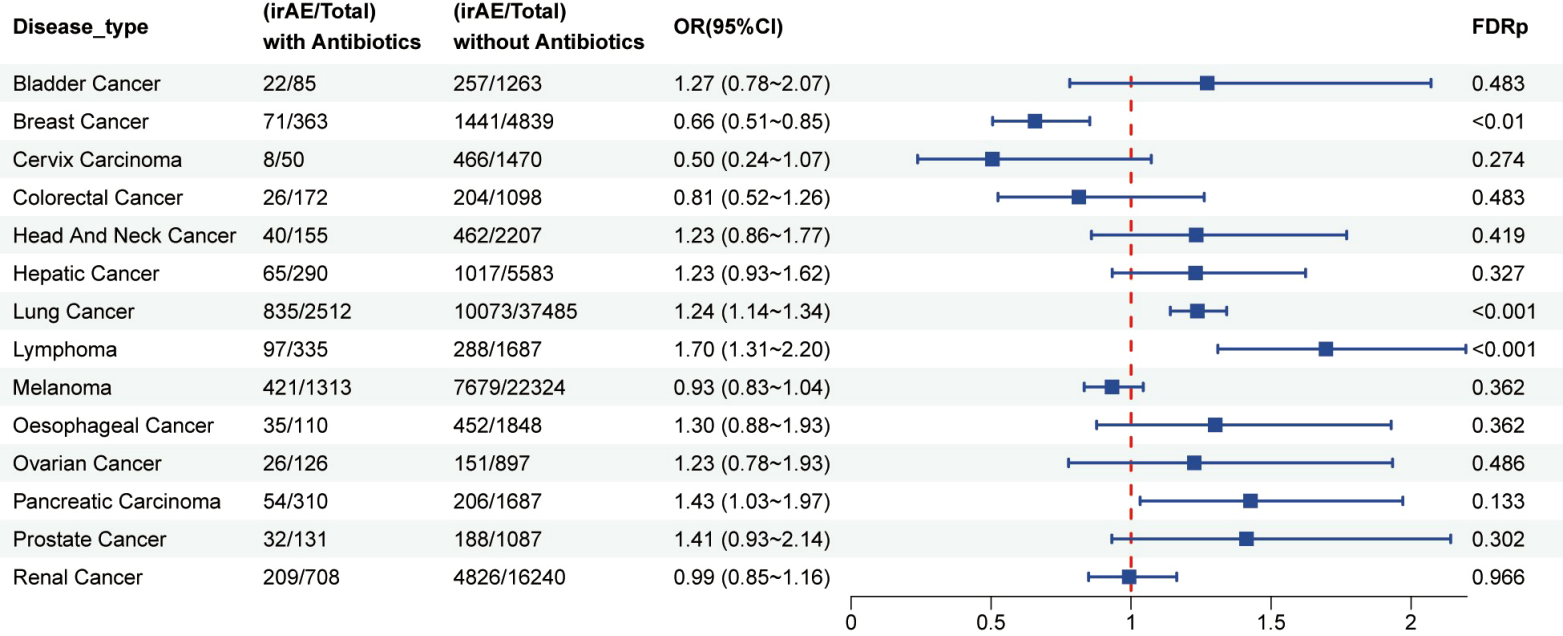
Forest plot illustrating the association between antibiotic use and irAEs across different cancer types in patients treated with ICIs.

The present retrospective study investigated whether antibiotic exposure influences the occurrence of irAEs in patients treated with ICIs. Multivariable logistic regression, with adjustments for age, sex, ICIs type, treatment regimen, and reporter country, revealed that antibiotic use was significantly correlated with a higher risk of irAEs (OR = 1.17; 95% CI: 1.12–1.23; FDR < 0.001). As illustrated in **Figure 2**, cancer-specific analyses showed an elevated risk in patients with lung cancer (OR = 1.24; 95% CI = 1.14–1.34; FDR < 0.001) and lymphoma (OR = 1.70; 95% CI: 1.31–2.20; FDR < 0.001), while a reduced risk was observed in breast cancer (OR = 0.66; 95% CI: 0.51–0.85; FDR < 0.01).

**Figure 3:**
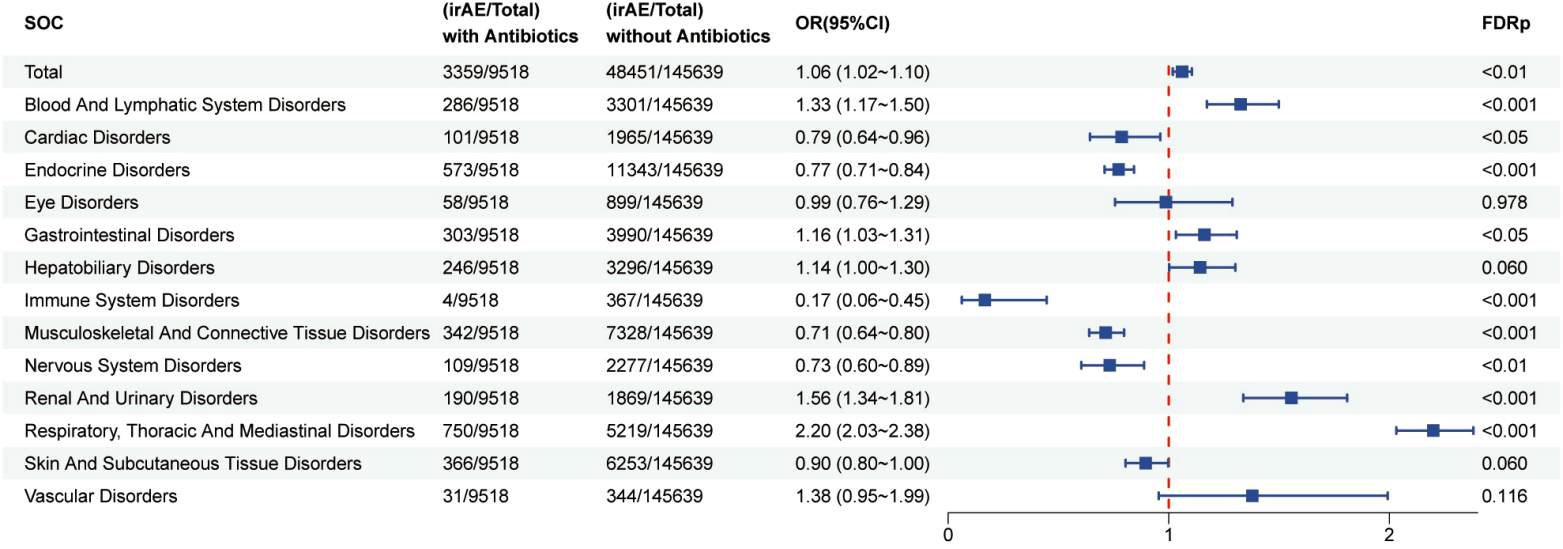
The forest plot illustrates the association between antibiotic use and irAEs across different organ systems in patients receiving ICIs therapy.

Organ-specific analyses categorized irAEs into 13 systems (**Figure 3**). Compared with non-users, patients receiving antibiotics had a greater likelihood of irAEs affecting the blood and lymphatic system (OR = 1.33; 95% CI: 1.17–1.51; FDR < 0.001), gastrointestinal system (OR = 1.16; 95% CI, 1.03–1.31; FDR < 0.05), renal and urinary system (OR = 1.56; 95% CI: 1.34–1.81; FDR < 0.001), hepatobiliary system (OR = 1.14; 95% CI: 1.00–1.31; FDR < 0.001), and the respiratory, thoracic and mediastinal system (OR = 2.20; 95% CI: 2.03–2.38; FDR < 0.001). In contrast, the incidence of cardiac, endocrine, immune, and nervous system irAEs appeared lower among antibiotic users.

### 3.3 Association between different classes of antibiotics and the occurrence of irAEs

**Figure 4:**
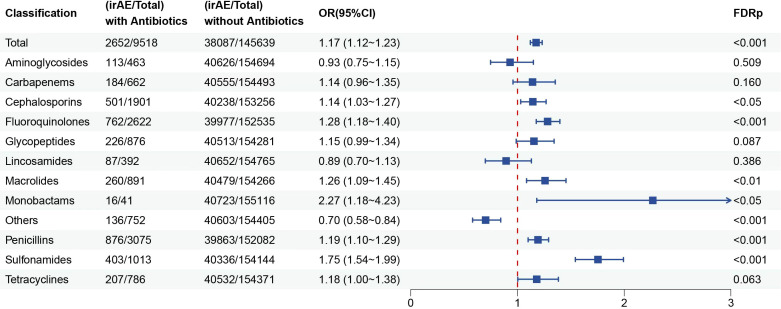
Multivariable logistic regression analysis of the association between the use of different classes of antibiotics in combination with ICIs and the risk of irAEs.

This study included twelve commonly used classes of antibiotics to evaluate their associations with the risk of irAEs in a broad patient population receiving ICIs therapy. Our analysis revealed that the use of macrolides (OR = 1.26, 95% CI: 1.09–1.45; FDR p < 0.01), fluoroquinolones (OR = 1.28, 95% CI: 1.18–1.40; FDR p< 0.001), sulfonamides (OR = 1.75, 95% CI: 1.54–1.99, FDR p < 0.001), cephalosporins (OR = 1.14 (1.03–1.27), FDR p < 0.05), monobactams (OR = 2.27 (1.18–4.23), FDR p < 0.05) and penicillins (OR = 1.19, 95% CI: 1.10–1.29; FDR p < 0.001) were significantly associated with an increased risk of developing irAEs (**Figure 4**). In contrast, no significant associations were observed for lincosamides, glycopeptides, carbapenems, or tetracyclines.

### 3.4 Association between the use of different classes of antibiotics and irAEs under various ICIs regimens

Previous studies have demonstrated that different ICIs exhibit distinct toxicity profiles, though the underlying reasons for these differences remain incompletely understood. This study aimed to evaluate the association between antibiotic use and the occurrence of irAEs across different ICIs treatment regimens, with a particular focus on comparing outcomes among patients receiving PD-1 inhibitors, PD-L1 inhibitors, and CTLA-4 inhibitors.

#### 3.4.1 Association between antibiotic use and irAEs in patients receiving PD-1 inhibitors

Among 99703 patients treated with PD-1 inhibitors,4672 had documented antibiotic use. As shown in **Supplementary Figure S1**, compared with non-users, only sulfonamide use was significantly associated with an increased overall risk of irAEs (OR = 1.62, 95% CI: 1.36–1.92; FDR p<0.001). We further evaluated the relationship between sulfonamide use and irAEs occurrence in patients receiving ICIs. Additionally, significant increases in irAEs risk were observed in the blood and lymphatic system, eye system, gastrointestinal system, hepatobiliary system, and respiratory, thoracic, and mediastinal system (**Supplementary Figure S2**). In addition, **Supplementary Figure S3** shows that sulfonamide use was linked to an increased risk of specific irAEs, including colitis (OR = 4.65, 95%CI: 3.41–6.35, FDR p<0.001), erythema multiforme (OR = 7.40, 95%CI: 4.13–13.27, FDR p<0.001), Pneumonia (OR = 2.65, 95%CI: 1.98–3.55, FDR p<0.001), Hepatitis (OR = 5.08, 95%CI: 4.13–13.27, FDR p<0.001), Thrombocytopenia (OR = 4.90, 95%CI: 2.17–11.07, FDR p<0.01).

#### 3.4.2 Association between antibiotic use and irAEs in patients receiving PD-L1 inhibitors

**Figure 5:**
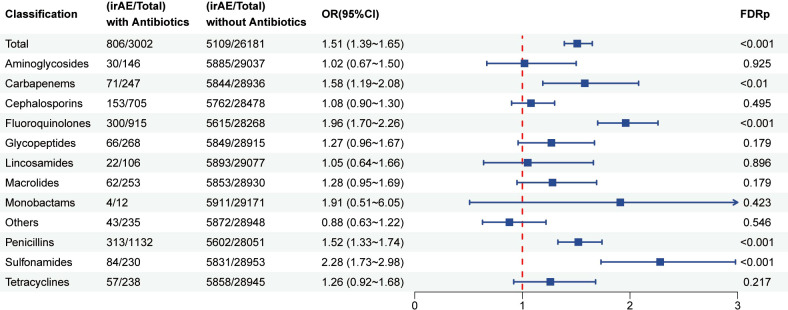
Multivariable logistic regression analysis of the association between the use of different classes of antibiotics in combination with PD-L1 inhibitors and the risk of irAEs.

**Figure 6:**
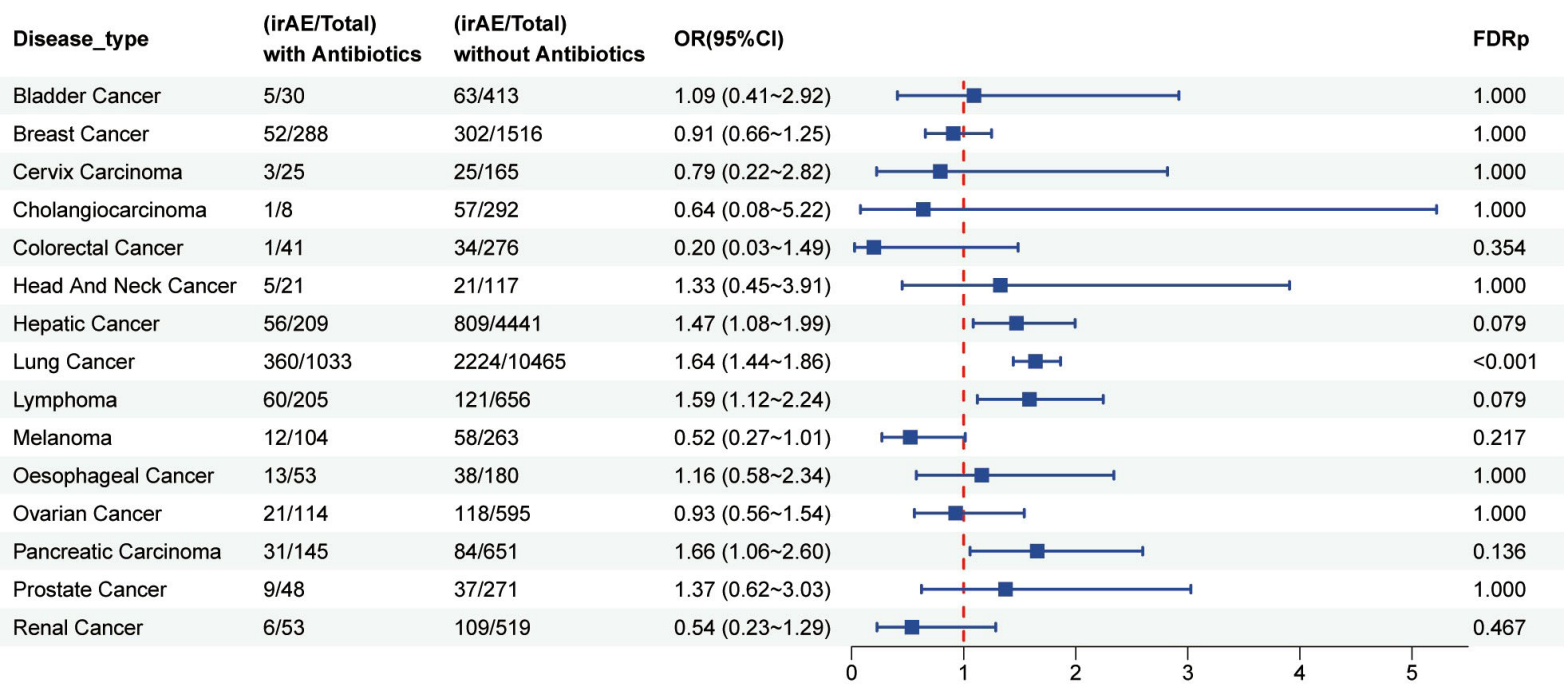
Forest plot illustrating the association between antibiotic use and irAEs across different cancer types in patients receiving PD-L1 inhibitor therapy.

**Figure 7:**
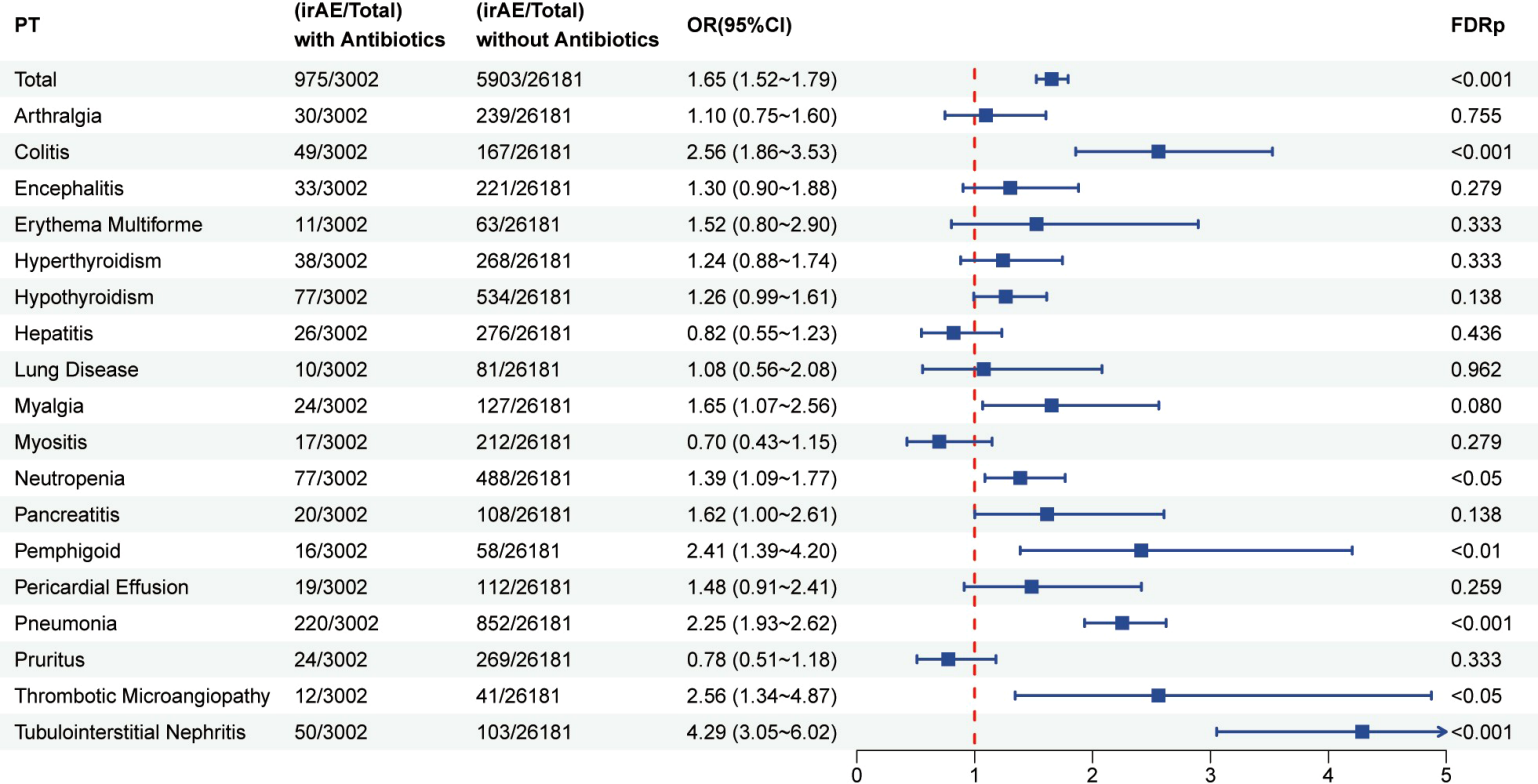
Forest plot illustrating the association between antibiotic use and various irAEs in patients treated with PD-L1 inhibitors.

A total of 29183 patients who received PD-L1 inhibitor therapy were included in this analysis, of whom 3002 had a history of antibiotic use. As shown in **Figure 5**, patients exposed to antibiotics exhibited a significantly increased risk of developing irAEs compared with non-users (OR = 1.51, 95% CI: 1.39–1.65; FDR p < 0.001). The elevated risk was particularly pronounced among users of carbapenems (OR = 1.58, 95% CI: 1.19–2.08; FDR p < 0.01), fluoroquinolones (OR = 1.96, 95% CI: 1.70–2.26; FDR p < 0.001), penicillin (OR = 1.52, 95% CI: 1.33–1.74; FDR p < 0.001), and sulfonamides (OR = 1.52, 95% CI: 1.33–1.74; FDR p < 0.001).Furthermore, as illustrated in **Figure 6**, antibiotic use was associated with a higher risk of irAEs among patients with lung cancer (OR = 1.68, 95% CI: 1.44–1.86; FDR p < 0.001). In addition, **Figure 7** and **Supplementary Figure S4** shows that antibiotic use was linked to an increased risk of specific irAEs, including colitis (OR = 2.56, 95% CI: 1.86–3.53; FDR p < 0.001), Pemphigoid (OR = 2.41, 95% CI: 1.39–4.20; FDR p < 0.05), neutropenic sepsis (OR = 10.36, 95% CI: 7.41–14.47; FDR p < 0.001), pneumonitis (OR = 2.25, 95% CI: 1.93–2.62; FDR p < 0.001), Thrombotic Microangiopathy (OR = 2.56, 95% CI: 1.34–4.87, FDR p < 0.05).

#### 3.4.3 Association between antibiotic use and irAEs in patients receiving CTLA-4 inhibitors

In the cohort of patients treated with CTLA-4 inhibitors, overall antibiotic use was not significantly associated with the risk of developing irAEs compared with non-users. However, the use of sulfonamide was strongly linked to an increased risk of irAEs (OR = 2.34, 95% CI: 1.79–3.07; FDR-adjusted p < 0.001) (**Supplementary Figure S5**).

### 3.5 Exploratory analysis of reported time to first irAE in patients receiving ICIs therapy

**Figure 8:**
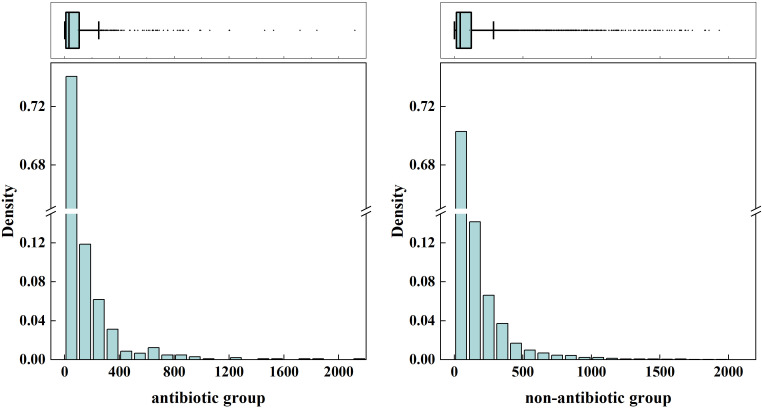
Box plots and histograms showing the distribution of reported time to first irAE among patients receiving ICIs therapy, comparing antibiotic co-reporting versus non-co-reporting groups.

**Figure 9:**
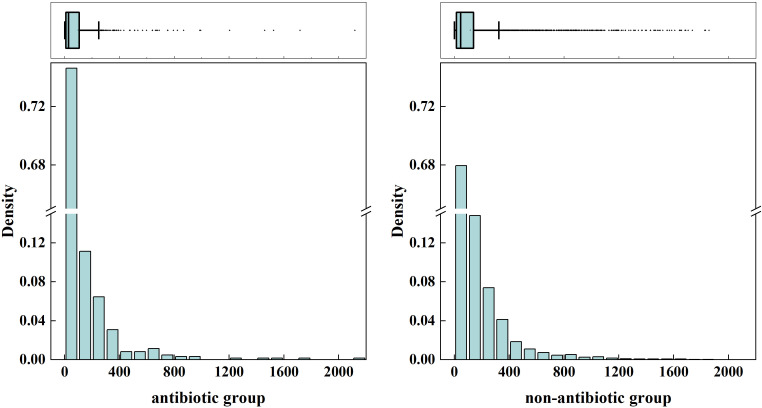
Box plots and histograms showing the distribution of reported time to first irAE among patients receiving PD-1 inhibitor therapy, comparing antibiotic co-reporting versus non-co-reporting groups. A shorter median reported time to first irAE was observed in the antibiotic co-reporting group.

To explore whether the temporal distribution of first reported irAEs differed between antibiotic co-reporting and non-co-reporting patients, we calculated the median and IQR of time from ICI treatment initiation to first irAE report for each group. Among all ICI-treated patients, the median time to first reported irAE was 31 days (IQR: 9–105 days) in the co-reporting group and 42 days (IQR: 14–122 days) in the non-exposed group. Box plots and histograms illustrating these distributions are presented in **Figure 8**. Stratified analyses by ICI type revealed that a similar pattern — with a shorter median time to first reported irAE in the antibiotic co-reporting group — was observed in patients receiving PD-1 inhibitors (antibiotic- co-reporting: 27 days (IQR:8–105); non-co-reporting: 45 days (IQR: 14–138)), but not in patients receiving PD-L1 or CTLA-4 inhibitors (**Figure 9**; **Supplementary Figures S6**, **S7**). These findings must be interpreted with caution. The observed difference in time to first reported irAE may reflect reporting artifacts, differential healthcare utilization, confounding by indication, or reverse causation — including the possibility that antibiotics were prescribed after irAE onset to treat secondary infections. The FAERS database does not provide verified treatment start dates, antibiotic initiation dates, or confirmation that antibiotic exposure preceded the irAE. Therefore, these temporal observations are presented as descriptive, exploratory findings only and do not support causal inference.

The antibiotic co-reporting group showed a shorter median time to first reported irAE [31 days (IQR: 9–105) vs. 42 days (IQR: 14–122); P < 0.001].

A shorter median reported time to first irAE was observed in the antibiotic co-reporting group [27 days (IQR: 8–105) vs. 45 days (IQR: 14–138); P < 0.001].

## 4 Discussion

In this study, we conducted a large-scale pharmacovigilance analysis using the FAERS database to explore the association between antibiotic co-reporting and irAEs in patients receiving ICIs therapy. Our analysis identified a safety signal suggesting that antibiotic co-reporting was associated with a higher reported frequency of irAEs. However, as a spontaneous reporting database analysis, these findings reflect associations in reported data and should not be interpreted as evidence of a causal relationship. This association held across multiple tumor types and ICIs regimens, with particularly notable effects in lung cancer and lymphoma patients, where the risk of ICIs-related pneumonitis was especially elevated. Although pneumonitis is one of the more frequent irAEs, its severe forms can greatly affect the course of treatment and even threaten patient survival (incidence in real-world settings reported between ~5% and 19%) (23). In addition, the magnitude of association differed across antibiotic classes, suggesting that different antibiotic categories may have heterogeneous relationships with irAEs. Our study found that PD-L1 inhibitors combined with antibiotics increase the risk of irAEs. One possible explanation for the differential association observed with PD-L1 inhibitors, drawn from the existing literature, relates to their distinct mechanism of action. We hypothesize that PD-L1 inhibitors directly act on tumor cells and antigen-presenting cells, whereas PD-1 inhibitors primarily target T cells (24, 25). Prior studies have suggested that antigen-presenting cells (APCs) are closely linked to gut microbiota signaling through pattern recognition receptors and metabolite sensing, which could theoretically make the PD-L1 pathway more susceptible to antibiotic-induced microbiome disruption (16). However, this interpretation is entirely speculative in the context of our study, as the FAERS database contains no microbiome data, no information on antibiotic duration, route, or dosing, and no confirmation that antibiotic exposure preceded irAE onset. Our study further revealed that combining PD-1 and CTLA-4 inhibitors with sulfonamide antibiotics increases the risk. Previous research indicates sulfonamides are typically regarded as broad-spectrum antimicrobials, exhibiting antibacterial activity against multiple Gram-positive and Gram-negative bacteria. Broad-spectrum antibiotics may more readily disrupt gut microbial community structure and diversity (26–28). Furthermore, sulfonamide drugs (e.g., sulfamethoxazole/trimethoprim) can trigger idiosyncratic hypersensitivity reactions and are associated with multiple immune-mediated diseases. We note that these reactions may synergize with ICI-induced immune activation (29–31). However, these mechanistic explanations remain speculative, as the FAERS database does not provide data on microbiome composition or immune cell populations.

Our exploratory descriptive analysis identified a pattern in which antibiotic co-reporting was associated with a shorter median reported time to first irAE among patients who experienced irAEs. However, this temporal observation is subject to the limitations of spontaneous reporting data described in the Methods section, including the inability to confirm whether antibiotic exposure preceded irAE onset. This finding does not establish that antibiotics accelerate irAE occurrence, as the observed difference may be attributable to greater disease severity and more intensive clinical monitoring in antibiotic-treated patients, reporting artifacts, differential healthcare utilization, confounding by indication, or reverse causation (e.g., antibiotics prescribed to treat infections arising from irAE-related immunosuppression) In summary, these findings highlight a pharmacovigilance signal suggesting that antibiotic co-reporting may be associated with both a higher reported frequency and an earlier reported onset of irAEs in ICI-treated patients. While these observations do not establish causality, they underscore the need for careful evaluation of antibiotic prescriptions in cancer patients receiving ICIs therapy to reduce the risk of potentially avoidable irAEs. In clinical practice, the necessity of antibiotic use should be rigorously assessed; if administration is deemed essential, patients should undergo enhanced monitoring throughout the entire course of ICIs treatment. Previous studies have demonstrated that patients receiving antibiotics before ICIs therapy exhibit a significantly increased risk of developing diarrhea and moderate-to-severe immune-related colitis (18).

The gut microbiota plays a crucial role in maintaining immune homeostasis, regulating T cell differentiation, and shaping anti-tumor immune responses (32, 33). Prior preclinical and observational studies have suggested that antibiotics can substantially reduce microbial diversity and deplete immunomodulatory commensals such as Akkermansia and Bifidobacterium, which may alter antigen presentation efficiency and T-cell activation thresholds (34). Furthermore, gut metabolites (such as short-chain fatty acids like butyrate) are essential for inducing regulatory T cell (Treg) differentiation, maintaining the intestinal epithelial barrier, and suppressing excessive inflammation (35) (36). Antibiotic-induced depletion of these metabolites weakens immune regulatory capacity and increases the risk of autoimmune-like responses (37). Animal studies suggest that fecal microbiota transplantation holds potential for modulating irAEs development (38). Exogenous probiotic supplements, such as Bifidobacterium, have also been demonstrated to improve colitis in mouse models treated with ICIs (39). Bifidobacteria modulate gut injury by altering gut microbiota composition in a Treg-dependent manner. Furthermore, this altered symbiotic flora enhances mitochondrial adaptability and IL-10-mediated inhibitory function of intestinal Treg cells, thereby contributing to improved colitis during immune checkpoint blockade. Bacteroides and Ruminococcus have also been identified as probiotics that help alleviate inflammation and disease progression (40). However, these findings from the literature provide a theoretical framework within which our pharmacovigilance signal might be contextualized. However, we emphasize that our study provides no direct evidence for microbiome-mediated mechanisms. The observed associations could equally be explained by confounding by indication (sicker patients receive more antibiotics and are monitored more closely), reverse causation (antibiotics prescribed after irAE-related complications), or surveillance bias (antibiotic users having more frequent clinical encounters). Future prospective cohort studies with comprehensive clinical and microbiome data are needed to validate these findings and elucidate potential mechanisms.

This study has several important limitations inherent to FAERS-based pharmacovigilance analyses. First, FAERS is a spontaneous reporting system subject to underreporting, reporting bias, stimulated reporting (e.g., following safety communications), and incomplete clinical information, which limits the accuracy of absolute incidence estimates and may introduce systematic distortions in association measures. Second, the observational nature of the study precludes causal inference. Critically, the covariates available for adjustment (age, sex, ICI type, treatment regimen, and reporter country) represent only a small subset of the relevant confounding structure. Unmeasured confounders including disease severity, cancer stage, line of therapy, ECOG performance status, corticosteroid and other immunosuppressive exposure, comorbidity burden, infection type and severity, reason for antibiotic prescription, and healthcare utilization patterns may have substantially influenced both antibiotic use and irAE reporting, leading to confounding by indication and surveillance bias. Third, the database does not systematically record the precise chronological sequence of drug administration, making it impossible to confirm whether antibiotic exposure preceded, was concurrent with, or followed irAE onset. This limitation is particularly consequential for the time-to-event analyses, which lack validated treatment start dates, standardized follow-up windows, and formal censoring definitions; accordingly, the survival analyses should be viewed as exploratory. Fourth, the substantial reduction in cases from initial download to final inclusion (as shown in the flow diagram) may introduce selection bias, as data missingness in FAERS is unlikely to be random. To assess this, we compared baseline characteristics between excluded and included cases, and additionally examined, within excluded cases, whether antibiotic users and non-users differed systematically (**Supplementary Tables 4**, **5**). The distribution of ICI type was nearly identical across all groups (CTLA-4: 17%; PD-1: 64–65%; PD-L1: 19%). However, excluded cases had higher rates of chemo-ICI regimens, irAEs, and reports from the United States. Importantly, within excluded cases, antibiotic co-reporting was associated with higher irAE rates (52% vs. 30%), directionally consistent with our primary findings. If selection bias exists, it most likely leads to an underestimation of the true antibiotic–irAE association, as the more severely affected patients were preferentially excluded. These findings should be interpreted with appropriate caution regarding their generalizability. Fifth, the FAERS database contains no microbiome data, antibiotic duration, dosing, route of administration, or inpatient/outpatient status, precluding any direct assessment of the hypothesized dysbiosis-mediated mechanisms. Future prospective studies should meticulously document medication timelines and incorporate microbiome profiling to elucidate how variations in antibiotic exposure influence the occurrence of irAEs.

## 5 Conclusion

Our pharmacovigilance analysis identified safety signals suggesting that antibiotic co-reporting may be associated with a higher reported frequency of irAEs and a shorter median time to first reported irAE among affected patients in those receiving ICIs. These findings should be interpreted as hypothesis-generating rather than confirmatory evidence of a causal relationship, given the inherent limitations of the FAERS database, including under-reporting, reporting bias, lack of exposure denominators, and inability to determine precise antibiotic exposure timing. The observed associations likely reflect complex interactions between antibiotics, gut microbiota, and host immune responses, though these mechanistic interpretations remain speculative without direct supporting data. Future prospective cohort studies integrating detailed medication timing records, microbiome profiling, and immunomic analyses are warranted to confirm these associations, elucidate underlying mechanisms, and guide rational antibiotic stewardship in patients undergoing immunotherapy.

## Data Availability

The raw data supporting the conclusions of this article will be made available by the authors, without undue reservation.
