# Supplementary material for: Antibiotic use and immune-related adverse events in patients treated with immune checkpoint inhibitors: analysis of the FAERS database
Source: Front Immunol. 2026 May 5;17:1733373. doi: 10.3389/fimmu.2026.1733373 (PMC13183842; doi:10.3389/fimmu.2026.1733373)
Supplement: Supplementary file 1 [file Table1.docx]

Supplementary Material

Antibiotic Use and Immune-Related Adverse Events in Patients Treated with Immune Checkpoint Inhibitors：analysis of the FAERS database

Jia Yu^1+^, Qinxiao Li^3+^, Shangpu Zou^4^, Yiyin Rong^3^, Yuting Zhang^1,2*^, Chengshui Chen^1,2*^

^1^Zhejiang Province Engineering Research Center for Endoscope Instruments and Technology Development, Department of Pulmonary and Critical Care Medicine, Quzhou People's Hospital,The Quzhou Affiliated Hospital of Wenzhou Medical University, Quzhou 324000, China.

^2^Key Laboratory of Interventional Pulmonology of Zhejiang Province, Department of Pulmonary and Critical Care Medicine, The First Affiliated Hospital of Wenzhou

Medical University, Wenzhou325000, China

^3^Department of Dermatology, Second Affiliated Hospital of Xi’an Jiaotong University, Xi'an, 710004, China.

^4^Department of Respiratory and Critical Care Medicine, The First Affiliated Hospital of Wenzhou Medical University, Wenzhou 325035, China.

*** Correspondence:**

Yuting Zhang: Email: [zhangyuting69@wmu.edu.cn](mailto:zhangyuting69@wmu.edu.cn)
Chengshui Chen:Email:chenchengshui@wmu.edu.cn. Fax:+86-577-55578186.

# Supplementary Figures and Tables

1.Supplementary Figures 1:STROBE Statement—Checklist of Items for Cross-Sectional Studies

| Item No | Recommendation | Page/Line No | Comment |
| --- | --- | --- | --- |
| Title and  Abstract | | | |
| 1a | Indicate the study's design with a commonly used term in the title or the abstract | Abstract | Cross-sectional pharmacovigilance study using the FAERS database |
| 1b | Provide in the abstract an informative and balanced  summary of what was done and what was found | Abstract | Background, methods, key results (OR=1.17), and conclusions are  provided |
| Introduction |  |  |  |
| 2 | Explain the scientiﬁc  background and rationale for the investigation being  reported | Introduction, Lines 99-110 | Background on ICIs, irAEs, gut microbiota, and gaps in current evidence described |
| 3 | State speciﬁc objectives, including any prespeciﬁed hypotheses | Introduction, Lines 111-119 | Objective: to evaluate association between antibiotic use and  risk/timing of irAEs in ICI-treated patients |
| Methods |  |  |  |
| 4 | Present key elements of study design early in the paper | Methods 2.1, Line 267-270 | Cross-sectional study using FAERS database; STROBE adherence  stated |
| 5 | Describe the setting,  locations, and relevant dates | Methods 2.2,  Lines 271-275 | FAERS database, 2014 to Q4 2024; FDA approval dates of each ICI  through end of 2024 |
| 6a | Give the eligibility criteria,  and the sources and methods of selection of participants | Methods 2.3,  Lines 276-285 | Patients receiving ICIs (PD-1, PD- L1, CTLA-4 inhibitors) identiﬁed from FAERS; antibiotic exposure deﬁned |
| 6b | For matched studies, give  matching criteria and number of exposed and unexposed | Table 1 | 9,518 antibiotic-exposed; 145,639 non-exposed |
| 7 | Clearly deﬁne all outcomes, exposures, predictors, | Methods 2.2-2.3 | Outcome: irAEs (deﬁned per  MedDRA); Exposure: 12 antibiotic classes; Confounders: age, sex, ICI |

|  | potential confounders, and eﬀect modiﬁers |  | type, treatment regimen, reporter country |
| --- | --- | --- | --- |
| 8 | For each variable of interest, | Methods 2.3, | FAERS database; MedDRA Version |
|  | give sources of data and | Supplementary | 27.1 for adverse event coding; |
|  | details of methods of | Tables 2-3 | antibiotic search terms in |
|  | assessment |  | Supplementary Table 2 |
| 9 | Describe any eﬀorts to | Methods 2.4; | Multivariable adjustment; FDR |
|  | address potential sources of | Discussion | correction for multiple testing; |
|  | bias | (Limitations) | limitations acknowledged |
| 10 | Explain how the study size was arrived at | Methods 2.3, Figure 1 | All eligible ICI-treated patients in FAERS during study period;  N=155,157 |
| 11 | Explain how quantitative | Methods 2.3; | Age categorized into groups (0-17, |
|  | variables were handled in the | Table 1 | 18-64, 65-75, ≥75); continuous |
|  | analyses |  | age also reported with SD |
| 12a | Describe all statistical  methods, including those used to control for  confounding | Methods 2.4,  Lines 351-357 | Multivariable logistic regression  adjusting for age, sex, ICI type,  treatment regimen, reporter country |
| 12b | Describe any methods used | Methods 2.3; | Subgroup analyses by cancer type, |
|  | to examine subgroups and | Results 3.3-3.5 | organ system, antibiotic class, and |
|  | interactions |  | ICI regimen |
| 12c | Explain how missing data | Methods 2.3 | Not explicitly stated (limitation of |
|  | were addressed |  | FAERS) |
| 12d | If applicable, explain how loss to follow-up was  addressed | N/A | Cross-sectional design; no follow- up |
| 12e | Describe any sensitivity analyses | Results 3.5 | Cox proportional hazards  regression for time-to-event analysis |
| Results | | | |
| 13a Report numbers of  individuals at each stage of study | | Figure 1; Results 3.1 | 155,157 total; 9,518 antibiotic- exposed; ﬂow diagram provided |

|  | potential confounders, and eﬀect modiﬁers |  | type, treatment regimen, reporter country |
| --- | --- | --- | --- |
| 8 | For each variable of interest, | Methods 2.3, | FAERS database; MedDRA Version |
|  | give sources of data and | Supplementary | 27.1 for adverse event coding; |
|  | details of methods of | Tables 2-3 | antibiotic search terms in |
|  | assessment |  | Supplementary Table 2 |
| 9 | Describe any eﬀorts to | Methods 2.4; | Multivariable adjustment; FDR |
|  | address potential sources of | Discussion | correction for multiple testing; |
|  | bias | (Limitations) | limitations acknowledged |
| 10 | Explain how the study size was arrived at | Methods 2.3, Figure 1 | All eligible ICI-treated patients in FAERS during study period;  N=155,157 |
| 11 | Explain how quantitative | Methods 2.3; | Age categorized into groups (0-17, |
|  | variables were handled in the | Table 1 | 18-64, 65-75, ≥75); continuous |
|  | analyses |  | age also reported with SD |
| 12a | Describe all statistical  methods, including those used to control for  confounding | Methods 2.4,  Lines 146-152 | Multivariable logistic regression  adjusting for age, sex, ICI type,  treatment regimen, reporter country |
| 12b | Describe any methods used | Methods 2.3; | Subgroup analyses by cancer type, |
|  | to examine subgroups and | Results 3.3-3.5 | organ system, antibiotic class, and |
|  | interactions |  | ICI regimen |
| 12c | Explain how missing data | Methods 2.3 | Not explicitly stated (limitation of |
|  | were addressed |  | FAERS) |
| 12d | If applicable, explain how loss to follow-up was  addressed | N/A | Cross-sectional design; no follow- up |
| 12e | Describe any sensitivity analyses | Results 3.5 | Cox proportional hazards  regression for time-to-event analysis |
| Results | | | |
| 13a Report numbers of  individuals at each stage of study | | Figure 1; Results 3.1 | 155,157 total; 9,518 antibiotic- exposed; ﬂow diagram provided |

| 18 | Summarise key results with reference to study objectives | Discussion, para 1 | Antibiotic exposure associated with increased irAE risk and delayed  onset |
| --- | --- | --- | --- |
| 19 | Discuss limitations of the  study, taking into account sources of potential bias or imprecision | Discussion,  Limitations para | FAERS limitations: underreporting, reporting bias, incomplete data,  inability to determine antibiotic timing, no causality inference |
| 20 | Give a cautious overall interpretation of results | Discussion;  Conclusion | Findings interpreted as hypothesis- generating; prospective studies  recommended |
| 21 | Discuss the generalisability  (external validity) of the study results | Discussion | Multi-country data from FAERS; predominantly US and Japan  reports |
| Other  Information |  |  |  |
| 22 | Give the source of funding and the role of the funders | Funding  statement | This study was supported by Zhejiang Provincial Key Research and Development Program 2020C03067 (C Chen) |

**Supplementary Table 2: Antibiotic Search Terms and Classification in the FAERS Database.**

| **Antibiotic Class** | **Specific Drug Names (Search Terms)** |
| --- | --- |
| Aminoglycosides | Gentamicin, Gentacycol, Gentamicin Sulfate, Sulfate, Gentamicin, Gentamicin Sulfate (USP), Garamycin, G-Myticin, GMyticin, G Myticin, Gentavet, Genticin, Gentamicin, Gentamycin, Amikacin Sulfate, Sulfate, Amikacin, BB-K8, BBK8, BB K8, BB-K 8, BB K 8, BBK 8, A.M.K, Amikacina Medical, Medical, Amikacina, Amikacina Normon, Normon, Amikacina, Amikafur, Amikalem, Amikason's, Amikayect, Amikin, Amiklin, Amukin, Biclin, Biklin, Gamikal, Kanbine, Oprad, Yectamid, Nebramycin Factor 6, Brulamycin, Tobramycin Sulfate, Sulfate, Tobramycin, Tobracin, Obracin, Nebcin, Nebicin, Streptomycin Sulfate, Streptomycin Sulphate, Streptomycin Sulfate (2:3) Salt, Estreptomicina CEPA, Estreptomicina Clariana, Estreptomicina Normon, Strepto-Fatol, Strepto Fatol, Strepto-Hefa, Strepto Hefa, Streptomycine Panpharma, Streptomycin Grünenthal, Neomycin Sulfate, Fradiomycin Sulfate, Neomycin Palmitate |
| Carbapenems | Merrem, SM 7338, SM-7338, SM7338, Ronem, Penem, 3-(5-Dimethylcarbamoylpyrrolidin-3-ylthio)-6-(1-hydroxyethyl)-4-methyl-7-oxo-1-azabicyclo (3.2.0) hept-2-ene-2-carboxylic acid, Imipemide, N-Formimidoylthienamycin, N Formimidoylthienamycin, Imipenem, Anhydrous, Anhydrous Imipenem, Imipenem Anhydrous, Anhydrous, Imipenem, MK-0787, MK 0787, MK0787, Ertapenem Sodium, Invanoz, Invanz |
| Cephalosporins | Cephazolin, Ancef, Totacef, Kefzol, Gramaxin, Cefazolin Sodium, Sodium, Cefazolin, Sodium Cephazolin, Cephazolin, Sodium, Cephazolin Sodium, Sodium, Cefazolin, Cefamezine, Cephamezine, Cefamedin, Cefazolin, Cefalexin, Cephalexin, (6r-(6alpha,7beta(s)))-Isomer, Cephalexin, Monosodium Salt, Cephalexin, Monosodium Salt, (6r-(6alpha,7beta))-Isomer, Cephalexin Monohydrochloride, Cephalexin Dihydride, Cephalexin, (6r-(6alpha,7alpha(r)))-Isomer, Cephalexin Hemihydrate, Cephalexin, (6r-(6alpha,7beta))-Isomer, Cephalexin Hydrochloride, Cephalexin Monohydrochloride, Monohydrate, Palitrex, Ceporexine, Cephalexin Monohydrate, 5-Thia-1-Azabicyclo(4.2.0)Oct-2-Ene-2-Carboxylic Acid, 7-((Aminophenylacetyl)Amino)-3-Methyl-8-Oxo-, (6r-(6alpha,7beta(R*)))-, Cephalexin, Cephuroxime, Ketocef, Zinacef, Cefuroxime, Cefatriaxone, Ceftriaxon, Ro13-9904, Ro139904, Ro13 9904, Ro-13-9904, Ro 13-9904, Ro 139904, Ro 13 9904, Ceftriaxone Sodium, Ceftriaxone Sodium, Anhydrous, Anhydrous Ceftriaxone Sodium, Rocephin, Rocefin, Rocefalin, Rocephine, Longacef, Longaceph, Lendacin, Tacex, Terbac, Benaxona, Cefaxona, Ceftrex, Ceftriaxone Irex, Ceftriaxona Ldp Torlan, Ceftriaxon Curamed, Ceftriaxon Hexal, Ceftriaxona Andreu, Ceftriaxone, Disodium Salt, Ceftriaxone, Disodium Salt, Hemiheptahydrate, Ceftriaxone, Cefotaxim, Cephotaxim, Cefotaxime Sodium, Sodium, Cefotaxime, Claforan, Klaforan, Primafen, Hr-756, Hr756, Hr 756, Ru-24756, Ru24756, Ru 24756, Cefradil, Benaxima, Biosint, Fotexina, Taporin, Kendrick, Cefotaxime, Cefepim, Bmy 28142, Bmy-28142, Bmy28142, Maxipime, Cefepime Hydrochloride, Quadrocef, Axépim, Cefepime, Ppi-0903, Ppi 0903, Ppi0903, Tak-599, Tak 599, Tak599, T 91825, 91825, T, T-91825, T91825, Ceftaroline Fosamil, Fosamil, Ceftaroline, Teflaro, Zinforo, Ceftaroline |
| Fluoroquinolones | Cipro, Bay-09867, Bay09867, Bay 09867, Ciprinol, Ciprofloxacin Hydrochloride, Hydrochloride, Ciprofloxacin, Ciprofloxacin Monohydrochloride Monohydrate, Monohydrate, Ciprofloxacin Monohydrochloride, Monohydrochloride Monohydrate, Ciprofloxacin, Ciprofloxacin Hydrochloride Anhydrous, Anhydrous, Ciprofloxacin Hydrochloride, Hydrochloride Anhydrous, Ciprofloxacin, Ofloxacin, (S)-Isomer, Quixin, Levaquin, Levofloxacin Anhydrous, Anhydrous, Levofloxacin, 1-Cyclopropyl-7-(2,8-diazabicyclo[4.3.0]non-8-yl)-6-fluoro-8-methoxy-1,4-dihydro-4-oxo-3-quinolinecarboxylic acid, BAY 12-8039, BAY 12 8039, BAY-128039, BAY128039, BAY 128039, BAY-12-8039, Moxifloxacin Hydrochloride, Avelox, Actira, Avalox, Izilox, Octegra, Proflox, Ofloxacine, Tarivid, Ofloxacin Hydrochloride, DL-8280, DL8280, DL 8280, Hoe-280, Hoe280, Hoe 280, ORF-28489, ORF28489, ORF 28489, Ru-43280, Ru43280, Ru 43280, DR-3355, DR3355, DR 3355 |
| Glycopeptides | Vancomycin Hydrochloride, Hydrochloride, Vancomycin, Vancocin, Vancocine, Vancocin HCl, Vancomycin Lilly, Vancomycin Sulfate, Sulfate, Vancomycin, Vancomycin Phosphate (1:2), Vancomycin Phosphate (1:2), Decahydrate, Diatracin, AB-Vancomycin, Vanco Azupharma, VANCO-cell, Vanco-saar, Vancomicina Abbott, Vancomicina Chiesi, Vancomicina Combino Phar, Vancomicina Norman, Vancomycin-ratiopharm, Vancomycin Hexal, Vancomycine Dakota, Teichomycin, Targocid, Teichomycin A2 |
| Lincosamides | 7-Chloro-7-deoxylincomycin, 7 Chloro 7 deoxylincomycin, Chlorlincocin, Chlolincocin, Clindamycin Hydrochloride, Hydrochloride, Clindamycin, Dalacin C, Cleocin, Clindamycin Monohydrochloride, Monohydrate, Monohydrate Clindamycin Monohydrochloride, Monohydrochloride, Monohydrate Clindamycin, Clindamycin Monohydrochloride, Monohydrochloride, Clindamycin |
| Macrolides | Erythromycin A,Erythromycin Lactate,Lactate, Erythromycin,Erythromycin Phosphate,Phosphate, Erythromycin,Erythromycin C,Ilotycin,Erycette,Erymax,T-Stat,TStat,T Stat,6-O-Methylerythromycin,TE-031,TE031,TE 031,Biaxin,A-56268,A56268,A 56268,Azythromycin,Zithromax,Ultreon,Zitromax,Azadose,Azitrocin,Azithromycin Dihydrate,Dihydrate, Azithromycin,Azithromycin Monohydrate,Monohydrate, Azithromycin,CP-62993,CP62993,CP 62993,Sumamed,Goxal,Toraseptol,Vinzam,Zentavion |
| Monobactams | Azthreonam, Az-threonam, Az threonam, Azactam, SQ-26,776, SQ 26,776, SQ26,776, Urobactam |
| Others | 2-Methyl-5-nitroimidazole-1-ethanol, 2 Methyl 5 nitroimidazole 1 ethanol, Metronidazole Hydrochloride, Clont, Danizol, Flagyl, Gineflavir, Metric, MetroGel, Metrodzhil, Metrogyl, Satric, Trichazol, Trichopol, Trivazol, Vagilen, Metronidazole Phosphate, Bayer 5360, Metronidazole Monohydrochloride, Metronidazole Phosphoester, Polymyxin B Sulfate, Aerosporin |
| Penicillins | Antibiotics, Penicillin, Penicillin Antibiotics, Penicillin, Penicillin, Amoxycillin, Amoxicilline, Amoxil, Amoxicillin, (R*)-Isomer, Amoxicillin Sodium, Amoxicillin Monosodium Salt, Amoxicillin Monopotassium Salt, Clamoxyl, Clamoxyl G.A., Penamox, Clamoxyl Parenteral, BRL-2333, BRL2333, BRL 2333, Wymox, Trimox, Polymox, Actimoxi, Amoxicillin Anhydrous, Amoxicillin Trihydrate, Hydroxyampicillin, Amoxicillin, Penicillin, Aminobenzyl, Aminobenzyl Penicillin, Aminobenzylpenicillin, Ukapen, Antibiotic KS-R1, Antibiotic KS R1, KS-R1, Antibiotic, Ampicillin Sodium, Sodium, Ampicillin, Ampicillin Trihydrate, Trihydrate, Ampicillin, Amcill, Omnipen, Polycillin, Pentrexyl, Ampicillin, Oxazocilline, Penicillin, Methylphenylisoxazolyl, Methylphenylisoxazolyl Penicillin, Prostaphlin, Oxacillin Sodium, Sodium, Oxacillin, Sodium Oxacillin, Oxacillin, Sodium, Oxacillin, Monosodium Salt, Anhydrous, Oxacillin, Monosodium Salt, Monohydrate, Oxacillin, Pipercillin, Pipracil, Pipril, Piperacillin Sodium, Sodium, Piperacillin, Piperacillin Monosodium Salt, Monosodium Salt, Piperacillin, Salt, Piperacillin Monosodium, Cl-227193, Cl227193, Cl 227193, Pipcil, Pipera-Hameln, Pipera Hameln, AB-Piperacillin, AB Piperacillin, Piperacillin Curasan, Curasan, Piperacillin, Piperacillin Fresenius, Piperacillin Hexal, T1220, T-1220, T 1220, Piperacillin-Ratiopharm, Piperacillin Ratiopharm, Piperacillin, Naphthamidopenicillin, Nafcil, Nafcillin Sodium, Sodium, Nafcillin, Sodium Nafcillin, Nafcillin, Sodium, Nafcillin, Monosodium Salt, Anhydrous, Nafcillin |
| Sulfonamides | Sulfamethylisoxazole, Sulfisomezole, Sulphamethoxazole, Gantanol, Trimpex, Proloprim |
| Tetracyclines | Topicycline, Achromycin V, Hostacyclin, Sustamycin, Tetrabid, Tetracycline Hydrochloride, Tetracycline Monohydrochloride, 4-Epitetracycline, 4 Epitetracycline, Achromycin, Doxycycline Monohydrate, Doxycycline-Chinoin, Doxycycline Chinoin, Hydramycin, Doxycycline Calcium, Vibramycin Novum, Vibramycin, Doxycycline Hyclate, Doxycycline Hemiethanolate, Doxycycline Monohydrochloride, 6-epimer, Doxycycline Monohydrochloride, 6 epimer, Doxycycline Phosphate (1:1), Doxycycline Monohydrochloride, Dihydrate, Doxycycline Calcium Salt (1:2), BMY-28689, BMY28689, BMY 28689, BU-3839T, BU3839T, BU 3839T, Vibravenos, Periostat, Atridox, Vibra-Tabs, Vibra Tabs, Doryx, Oracea, 2-Naphthacenecarboxamide, 4-(dimethylamino)-1,4,4a,5,5a,6,11,12a-octahydro-3,5,10,12,12a-pentahydroxy-6-methyl-1,11-dioxo-, (4S-(4alpha,4aalpha,5alpha,5aalpha,6alpha,12aalpha))-, Alpha-6-Deoxyoxytetracycline, Alpha 6 Deoxyoxytetracycline, Minocin, Minocin MR, Minocycline Hydrochloride, Hydrochloride, Minocycline, Minocycline Monohydrochloride, Monohydrochloride, Minocycline, Minocycline, (4R-(4 alpha,4a beta,5a beta,12a beta))-Isomer, Minox 50, Akamin, Akne-Puren, Akne Puren, Aknemin, Aknin-Mino, Aknin Mino, Aknosan, Apo-Minocycline, Apo Minocycline, Arestin, Dynacin, Lederderm, Blemix, Cyclomin, Cyclops, Dentomycin, Icht-Oral, Icht Oral, Klinomycin, Minakne, Mino-Wolff, Mino Wolff, Minoclir, Minolis, Minoplus, Mestacine, Minomycin, Minotab, Mynocine |

Supplementary Table 1 provides the complete list of antibiotic drug names and search terms used for data extraction from the FAERS database, organized by antibiotic class (aminoglycosides, carbapenems, cephalosporins, fluoroquinolones, glycopeptides, lincosamides, macrolides, monobactams, others, penicillins, sulfonamides, and tetracyclines).

**Supplementary Table 3** **:Selected Immune-Related Adverse Event Preferred Terms (irAEs) from the FAERS Database and Their Corresponding MedDRA (Version 27.1) System Organ Class (SOC).**

| **SOC** | **PT** |
| --- | --- |
| Blood And Lymphatic System Disorders | Acquired Haemophilia |
| Blood And Lymphatic System Disorders | Acquired Von Willebrand'S Disease |
| Blood And Lymphatic System Disorders | Antiphospholipid Syndrome |
| Blood And Lymphatic System Disorders | Aplasia Pure Red Cell |
| Blood And Lymphatic System Disorders | Aplastic Anaemia |
| Blood And Lymphatic System Disorders | Autoimmune Anaemia |
| Blood And Lymphatic System Disorders | Autoimmune Aplastic Anaemia |
| Blood And Lymphatic System Disorders | Autoimmune Haemolytic Anaemia |
| Blood And Lymphatic System Disorders | Autoimmune Neutropenia |
| Blood And Lymphatic System Disorders | Cyclic Neutropenia |
| Blood And Lymphatic System Disorders | Hypoplastic Anaemia |
| Blood And Lymphatic System Disorders | Immune Thrombocytopenia |
| Blood And Lymphatic System Disorders | Immune-Mediated Cytopenia |
| Blood And Lymphatic System Disorders | Neutropenia |
| Blood And Lymphatic System Disorders | Thrombotic Microangiopathy |
| Blood And Lymphatic System Disorders | Warm Autoimmune Haemolytic Anaemia |
| Blood And Lymphatic System Disorders | Neutropenic Infection |
| Blood And Lymphatic System Disorders | Neutropenic Sepsis |
| Cardiac Disorders | Autoimmune Myocarditis |
| Cardiac Disorders | Autoimmune Pericarditis |
| Cardiac Disorders | Cardiac Tamponade |
| Cardiac Disorders | Immune-Mediated Myocarditis |
| Cardiac Disorders | Immune-Mediated Pericarditis |
| Cardiac Disorders | Myocardial Fibrosis |
| Cardiac Disorders | Pericardial Effusion |
| Cardiac Disorders | Pericardial Effusion Malignant |
| Cardiac Disorders | Infective Pericardial Effusion |
| Cardiac Disorders | Meningitis Aseptic |
| Congenital, Familial And Genetic Disorders | Congenital Aplastic Anaemia |
| Congenital, Familial And Genetic Disorders | Congenital Hyperthyroidism |
| Congenital, Familial And Genetic Disorders | Congenital Hypogonadotropic Hypogonadism |
| Congenital, Familial And Genetic Disorders | Congenital Hypothyroidism |
| Congenital, Familial And Genetic Disorders | Familial Haemophagocytic Lymphohistiocytosis |
| Endocrine Disorders | Adrenal Insufficiency |
| Endocrine Disorders | Autoimmune Hypothyroidism |
| Endocrine Disorders | Autoimmune Thyroiditis |
| Endocrine Disorders | Central Hypothyroidism |
| Endocrine Disorders | Ectopic Hyperthyroidism |
| Endocrine Disorders | Hyperparathyroidism |
| Endocrine Disorders | Hyperparathyroidism Primary |
| Endocrine Disorders | Hyperparathyroidism Secondary |
| Endocrine Disorders | Hyperparathyroidism Tertiary |
| Endocrine Disorders | Hyperthyroidism |
| Endocrine Disorders | Hypogonadism |
| Endocrine Disorders | Hypogonadism Female |
| Endocrine Disorders | Hypogonadism Male |
| Endocrine Disorders | Hypopituitarism |
| Endocrine Disorders | Hypothyroidism |
| Endocrine Disorders | Immune-Mediated Adrenal Insufficiency |
| Endocrine Disorders | Immune-Mediated Endocrinopathy |
| Endocrine Disorders | Immune-Mediated Hyperthyroidism |
| Endocrine Disorders | Immune-Mediated Hypophysitis |
| Endocrine Disorders | Immune-Mediated Hypothyroidism |
| Endocrine Disorders | Immune-Mediated Thyroiditis |
| Endocrine Disorders | Lymphocytic Hypophysitis |
| Endocrine Disorders | Primary Adrenal Insufficiency |
| Endocrine Disorders | Primary Hyperthyroidism |
| Endocrine Disorders | Primary Hypogonadism |
| Endocrine Disorders | Primary Hypothyroidism |
| Endocrine Disorders | Secondary Hyperthyroidism |
| Endocrine Disorders | Secondary Hypogonadism |
| Endocrine Disorders | Tertiary Adrenal Insufficiency |
| Eye Disorders | Autoimmune Uveitis |
| Eye Disorders | Fuchs' Syndrome |
| Eye Disorders | Idiopathic Orbital Inflammation |
| Eye Disorders | Immune-Mediated Scleritis |
| Eye Disorders | Immune-Mediated Uveitis |
| Eye Disorders | Iritis |
| Eye Disorders | Orbital Myositis |
| Eye Disorders | Scleritis |
| Eye Disorders | Uveitis |
| Eye Disorders | Vogt-Koyanagi-Harada Disease |
| Eye Disorders | Conjunctivitis |
| Gastrointestinal Disorders | Acute Haemorrhagic Ulcerative Colitis |
| Gastrointestinal Disorders | Autoimmune Colitis |
| Gastrointestinal Disorders | Autoimmune Pancreatitis |
| Gastrointestinal Disorders | Colitis Ischaemic |
| Gastrointestinal Disorders | Colitis Microscopic |
| Gastrointestinal Disorders | Immune-Mediated Enterocolitis |
| Gastrointestinal Disorders | Immune-Mediated Gastritis |
| Gastrointestinal Disorders | Immune-Mediated Oesophagitis |
| Gastrointestinal Disorders | Immune-Mediated Pancreatitis |
| Gastrointestinal Disorders | Neutropenic Colitis |
| Gastrointestinal Disorders | Oral Lichen Planus |
| Gastrointestinal Disorders | Pancreatitis |
| Gastrointestinal Disorders | Terminal Ileitis |
| Hepatobiliary Disorders | Autoimmune Hepatitis |
| Hepatobiliary Disorders | Immune-Mediated Cholangitis |
| Hepatobiliary Disorders | Immune-Mediated Cholestasis |
| Hepatobiliary Disorders | Immune-Mediated Hepatic Disorder |
| Hepatobiliary Disorders | Immune-Mediated Hepatitis |
| Immune System Disorders | Eosinophilic Granulomatosis With Polyangiitis |
| Immune System Disorders | Immune-Mediated Adverse Reaction |
| Musculoskeletal And Connective Tissue Disorders | Infective Tenosynovitis |
| Musculoskeletal And Connective Tissue Disorders | Necrotising Fasciitis |
| Musculoskeletal And Connective Tissue Disorders | Tuberculous Tenosynovitis |
| Musculoskeletal And Connective Tissue Disorders | Antisynthetase Syndrome |
| Musculoskeletal And Connective Tissue Disorders | Arthralgia |
| Musculoskeletal And Connective Tissue Disorders | Arthritis |
| Musculoskeletal And Connective Tissue Disorders | Autoimmune Myositis |
| Musculoskeletal And Connective Tissue Disorders | Dermatomyositis |
| Musculoskeletal And Connective Tissue Disorders | Eosinophilic Fasciitis |
| Musculoskeletal And Connective Tissue Disorders | Fasciitis |
| Musculoskeletal And Connective Tissue Disorders | Immune-Mediated Arthritis |
| Musculoskeletal And Connective Tissue Disorders | Immune-Mediated Myositis |
| Musculoskeletal And Connective Tissue Disorders | Juvenile Psoriatic Arthritis |
| Musculoskeletal And Connective Tissue Disorders | Myalgia |
| Musculoskeletal And Connective Tissue Disorders | Myositis |
| Musculoskeletal And Connective Tissue Disorders | Oligoarthritis |
| Musculoskeletal And Connective Tissue Disorders | Plantar Fasciitis |
| Musculoskeletal And Connective Tissue Disorders | Polyarthritis |
| Musculoskeletal And Connective Tissue Disorders | Polymyalgia Rheumatica |
| Musculoskeletal And Connective Tissue Disorders | Polymyositis |
| Musculoskeletal And Connective Tissue Disorders | Psoriatic Arthropathy |
| Musculoskeletal And Connective Tissue Disorders | Rheumatoid Arthritis |
| Musculoskeletal And Connective Tissue Disorders | Spondyloarthropathy |
| Musculoskeletal And Connective Tissue Disorders | Systemic Lupus Erythematosus |
| Musculoskeletal And Connective Tissue Disorders | Tenosynovitis |
| Musculoskeletal And Connective Tissue Disorders | Tenosynovitis Stenosans |
| Nervous System Disorders | Encephalitis |
| Nervous System Disorders | Axonal Neuropathy |
| Nervous System Disorders | Chronic Inflammatory Demyelinating Polyradiculoneuropathy |
| Nervous System Disorders | Demyelinating Polyneuropathy |
| Nervous System Disorders | Encephalitis Autoimmune |
| Nervous System Disorders | Immune-Mediated Encephalitis |
| Nervous System Disorders | Immune-Mediated Encephalopathy |
| Nervous System Disorders | Immune-Mediated Myasthenia Gravis |
| Nervous System Disorders | Immune-Mediated Neurological Disorder |
| Nervous System Disorders | Immune-Mediated Neuropathy |
| Nervous System Disorders | Lower Motor Neurone Lesion |
| Nervous System Disorders | Mononeuropathy Multiplex |
| Nervous System Disorders | Motor Neurone Disease |
| Nervous System Disorders | Multifocal Motor Neuropathy |
| Nervous System Disorders | Myasthenia Gravis |
| Nervous System Disorders | Myasthenia Gravis Crisis |
| Nervous System Disorders | Myasthenia Gravis Neonatal |
| Nervous System Disorders | Myelitis Transverse |
| Nervous System Disorders | Optic Neuritis |
| Nervous System Disorders | Peripheral Motor Neuropathy |
| Nervous System Disorders | Peripheral Sensorimotor Neuropathy |
| Nervous System Disorders | Sensorimotor Disorder |
| Nervous System Disorders | Subacute Inflammatory Demyelinating Polyneuropathy |
| Nervous System Disorders | Upper Motor Neurone Lesion |
| Pregnancy, Puerperium And Perinatal Conditions | Neonatal Thyrotoxicosis |
| Renal And Urinary Disorders | Glomerulonephritis |
| Renal And Urinary Disorders | Glomerulonephritis Acute |
| Renal And Urinary Disorders | Glomerulonephritis Chronic |
| Renal And Urinary Disorders | Glomerulonephritis Membranous |
| Renal And Urinary Disorders | Glomerulonephritis Proliferative |
| Renal And Urinary Disorders | Glomerulonephritis Rapidly Progressive |
| Renal And Urinary Disorders | Immune-Mediated Cystitis |
| Renal And Urinary Disorders | Immune-Mediated Nephritis |
| Renal And Urinary Disorders | Immune-Mediated Renal Disorder |
| Renal And Urinary Disorders | Lupus Nephritis |
| Renal And Urinary Disorders | Renal Tubular Acidosis |
| Renal And Urinary Disorders | Tubulointerstitial Nephritis |
| Renal And Urinary Disorders | Tubulointerstitial Nephritis And Uveitis Syndrome |
| Respiratory, Thoracic And Mediastinal Disorders | Pneumonia |
| Respiratory, Thoracic And Mediastinal Disorders | Pneumonia Aspiration |
| Respiratory, Thoracic And Mediastinal Disorders | Alveolitis |
| Respiratory, Thoracic And Mediastinal Disorders | Combined Pulmonary Fibrosis And Emphysema |
| Respiratory, Thoracic And Mediastinal Disorders | Idiopathic Pulmonary Fibrosis |
| Respiratory, Thoracic And Mediastinal Disorders | Immune-Mediated Lung Disease |
| Respiratory, Thoracic And Mediastinal Disorders | Pulmonary Fibrosis |
| Respiratory, Thoracic And Mediastinal Disorders | Pulmonary Haemorrhage |
| Respiratory, Thoracic And Mediastinal Disorders | Pulmonary Haemorrhage Neonatal |
| Respiratory, Thoracic And Mediastinal Disorders | Pulmonary Sarcoidosis |
| Respiratory, Thoracic And Mediastinal Disorders | Pulmonary Vasculitis |
| Skin And Subcutaneous Tissue Disorders | Alopecia Areata |
| Skin And Subcutaneous Tissue Disorders | Alopecia Universalis |
| Skin And Subcutaneous Tissue Disorders | Angiodermatitis |
| Skin And Subcutaneous Tissue Disorders | Anogenital Lichen Planus |
| Skin And Subcutaneous Tissue Disorders | Cutaneous Sarcoidosis |
| Skin And Subcutaneous Tissue Disorders | Cutaneous Vasculitis |
| Skin And Subcutaneous Tissue Disorders | Dermatitis Herpetiformis |
| Skin And Subcutaneous Tissue Disorders | Erythema Multiforme |
| Skin And Subcutaneous Tissue Disorders | Erythema Nodosum |
| Skin And Subcutaneous Tissue Disorders | Granuloma Annulare |
| Skin And Subcutaneous Tissue Disorders | Immune-Mediated Dermatitis |
| Skin And Subcutaneous Tissue Disorders | Lichen Planopilaris |
| Skin And Subcutaneous Tissue Disorders | Lichen Planus |
| Skin And Subcutaneous Tissue Disorders | Lichen Planus Pemphigoides |
| Skin And Subcutaneous Tissue Disorders | Neuropathic Pruritus |
| Skin And Subcutaneous Tissue Disorders | Panniculitis |
| Skin And Subcutaneous Tissue Disorders | Parapsoriasis |
| Skin And Subcutaneous Tissue Disorders | Pemphigoid |
| Skin And Subcutaneous Tissue Disorders | Pemphigus |
| Skin And Subcutaneous Tissue Disorders | Pruritus |
| Skin And Subcutaneous Tissue Disorders | Psoriasis |
| Skin And Subcutaneous Tissue Disorders | Pustular Psoriasis |
| Skin And Subcutaneous Tissue Disorders | Pyoderma Gangrenosum |
| Skin And Subcutaneous Tissue Disorders | Rash Pruritic |
| Skin And Subcutaneous Tissue Disorders | Rebound Psoriasis |
| Skin And Subcutaneous Tissue Disorders | Subacute Cutaneous Lupus Erythematosus |
| Skin And Subcutaneous Tissue Disorders | Vitiligo |
| Vascular Disorders | Cryoglobulinaemia |
| Vascular Disorders | Giant Cell Arteritis |
| Vascular Disorders | Vasculitis |

**Supplementary Table 4. Comparison of baseline characteristics between included and excluded cases.**

| **Characteristic** | **Included Cases (N=155,157)** | **Excluded Cases** |
| --- | --- | --- |
| **Antibiotic use** |  |  |
| **Without antibiotic** | **145,639 (93.9%)** | **109,705 (97%)**¹ |
| **With antibiotic** | **9,518 (6.1%)** | **2909 (2.6%)**¹ |
| **Age, mean ± SD** | **65 ± 14** | **65 ± 18²** |
| **Available N for age** | **155,157** | **30,832** |
| **Age group** |  |  |
| **0–17** | **1,985 (1.3%)** | **1,465 (4.8%)** |
| **18–64** | **70,444 (45%)** | **13,172 (43%)** |
| **65–75** | **57,843 (37%)** | **10,984 (36%)** |
| **≥75** | **24,868 (16%)** | **5,211 (17%)** |
| **Sex** |  |  |
| **Available N for sex** | **155,157** | **78,288** |
| **Female** | **60,032 (39%)** | **33,811 (43%)** |
| **Male** | **95,125 (61%)** | **44,477 (57%)** |
| **ICIs type** |  |  |
| **Available N** | **155,157** | **112,614** |
| **CTLA-4** | **26,271 (17%)** | **18,833 (17%)** |
| **PD-1** | **99,703 (64%)** | **72,553 (64%)** |
| **PD-L1** | **29,183 (19%)** | **21,228 (19%)** |
| **Treatment regimen** |  |  |
| **Available N** | **155,157** | **93,278** |
| **Monotherapy** | **32,729 (21%)** | **7,393 (8%)** |
| **Multi-ICI** | **90,290 (58%)** | **68,195 (73%)** |
| **Chemo-ICI** | **32,138 (21%)** | **17,690 (19%)** |
| **Reporter region** |  |  |
| **Available N** | **155,157** | **112,614** |
| **United States** | **42,633 (27%)** | **46,870 (42%)** |
| **Japan** | **34,432 (22%)** | **24,254 (22%)** |
| **China** | **6,683 (4.3%)** | **5,408 (4.8%)** |
| **Europe³** | **38,302 (25%)** | **23,917 (21%)** |
| **Other** | **33,107 (21%)** | **12,165 (11%)** |
| **irAEs** |  |  |
| **Available N** | **155,157** | **111,784** |
| **No** | **114,418 (74%)** | **77,120 (69%)** |
| **Yes** | **40,739 (26%)** | **34,664 (31%)** |

**Abbreviations: ICIs, immune checkpoint inhibitors; irAEs, immune-related adverse events; SD, standard deviation.**

¹ Excluded cases were primarily excluded due to missing data on age, sex, drug usage, adverse reaction type, or indication. The available N for each variable differs because different cases had different patterns of missing data. Percentages are calculated based on available N for each variable.

² Age statistics for excluded cases are based on the subset with non-missing age data (N=30,832), representing only 27% of excluded cases. The larger SD may reflect a different age distribution or data quality issues among partially complete reports.

³ For included cases, Europe includes France, Germany, Italy, United Kingdom, and Spain as reported in Table 1. For excluded cases, Europe is reported as a combined category from the original analysis.

**Supplementary Table 5.** Baseline characteristics of excluded cases stratified by antibiotic co-reporting.

| **Characteristic** | **Without antibiotic (n=29,311–109,705)** | **With antibiotic (n=1,521–2,909)** | **p-value** |
| --- | --- | --- | --- |
| **Age, mean ± SD** | 65 ± 18 | 66 ± 14 | <0.001 |
| **Age group** |  |  | <0.001 |
| 0–17 | 1,435 (4.9%) | 30 (2.0%) |  |
| 18–64 | 12,506 (43%) | 666 (44%) |  |
| 65–75 | 10,361 (35%) | 623 (41%) |  |
| ≥75 | 5,009 (17%) | 202 (13%) |  |
| **Sex** |  |  | <0.001 |
| Female | 32,854 (43%) | 957 (39%) |  |
| Male | 43,012 (57%) | 1,465 (61%) |  |
| **ICI type** |  |  | <0.001 |
| CTLA-4 | 18,211 (17%) | 622 (21%) |  |
| PD-1 | 70,965 (65%) | 1,588 (55%) |  |
| PD-L1 | 20,529 (19%) | 699 (24%) |  |
| **Reporter region** |  |  | <0.001 |
| United States | 46,100 (42%) | 770 (27%) |  |
| China | 5,343 (4.9%) | 65 (2.2%) |  |
| Japan | 23,803 (22%) | 451 (15%) |  |
| Europe | 22,688 (21%) | 1,229 (42%) |  |
| Other | 11,771 (11%) | 394 (13%) |  |
| **Treatment regimen** |  |  | <0.001 |
| Chemo-ICI | 16,766 (18%) | 924 (40%) |  |
| Dual-ICI | 66,933 (74%) | 1,262 (54%) |  |
| Monotherapy | 7,255 (8.0%) | 138 (5.9%) |  |
| **irAEs** |  |  | <0.001 |
| No | 75,746 (70%) | 1,374 (48%) |  |
| Yes | 33,160 (30%) | 1,504 (52%) |  |

*Abbreviations: ICI, immune checkpoint inhibitor; irAE, immune-related adverse event; SD, standard deviation. Available N varies by variable due to missing data patterns in excluded cases.*

## Supplementary Figures


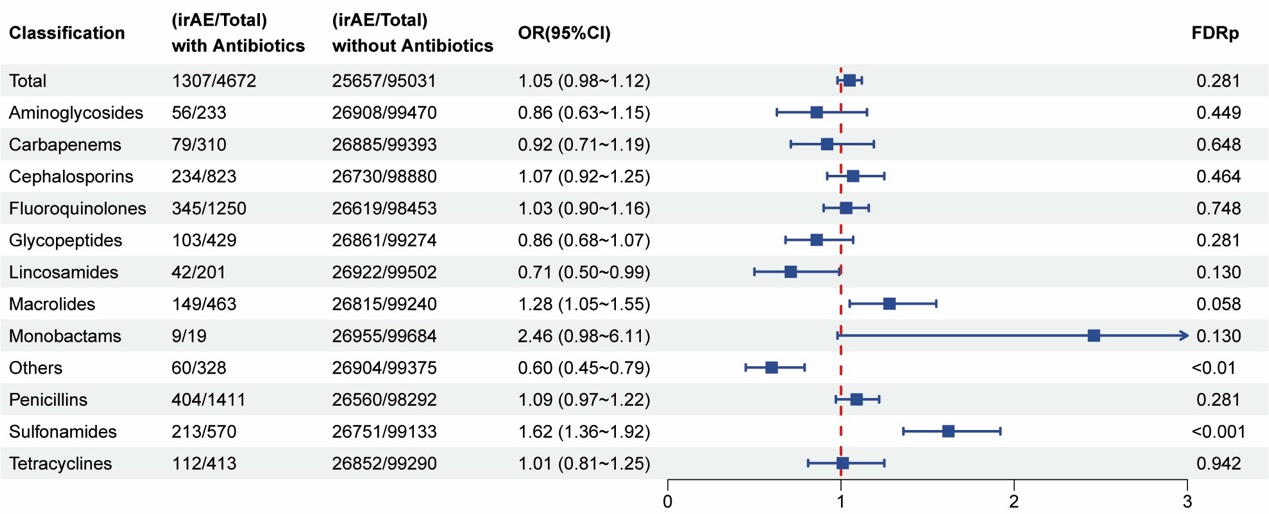


Figure S1: Multivariable logistic regression analysis of the association between the use of different classes of antibiotics in combination with PD-1 inhibitors and the risk of irAEs. The p-values are adjusted using the false discovery rate (FDR). irAEs: immune-related adverse events, ICIs:immune checkpoint inhibitors


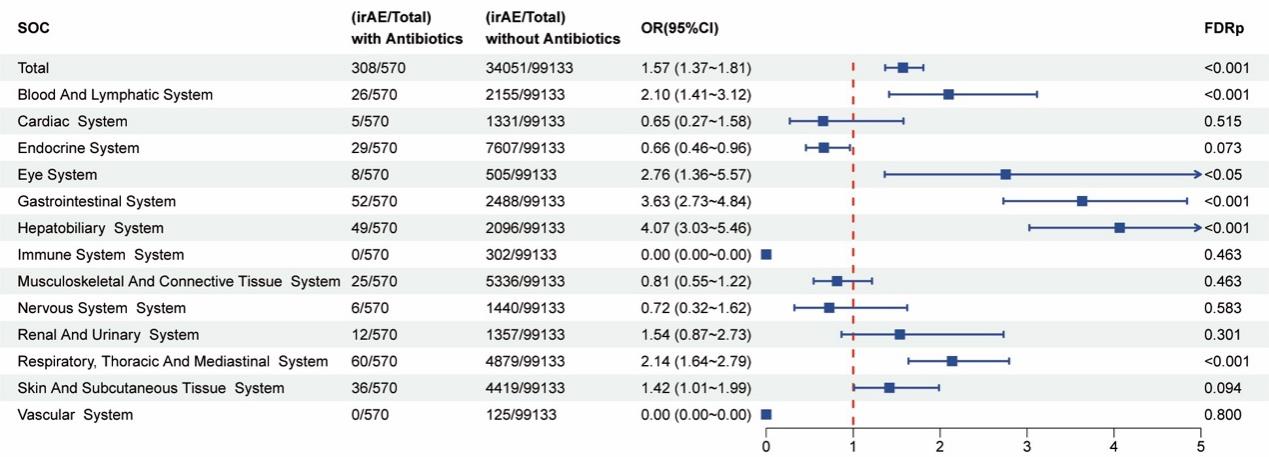


Figure S2: Forest plot illustrating the association between sulfonamide use and various irAEs in patients treated with PD-1 inhibitors.


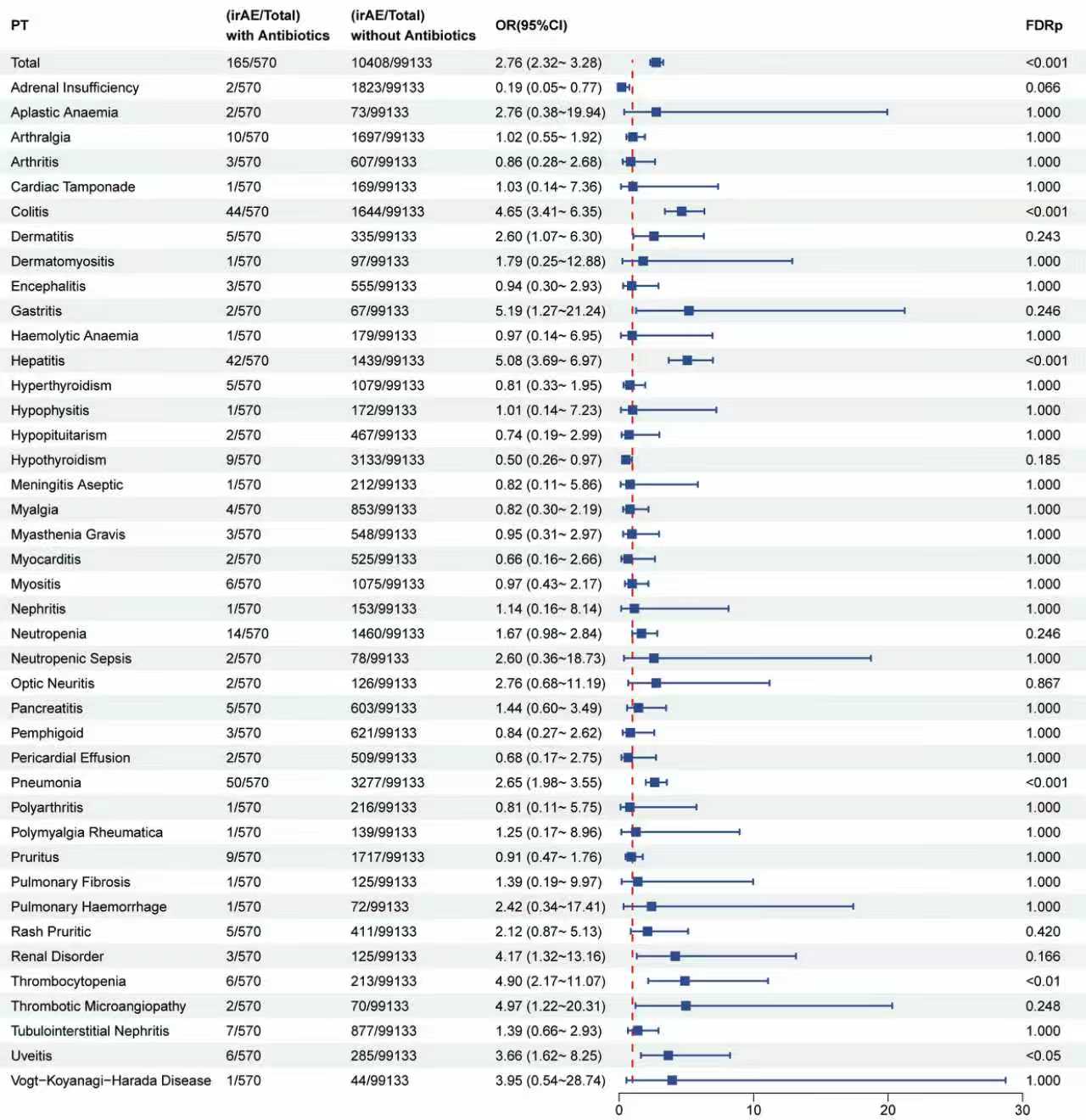


Figure S3: Forest plot illustrating the association between sulfonamide use and various irAEs in patients treated with PD-1 inhibitors.


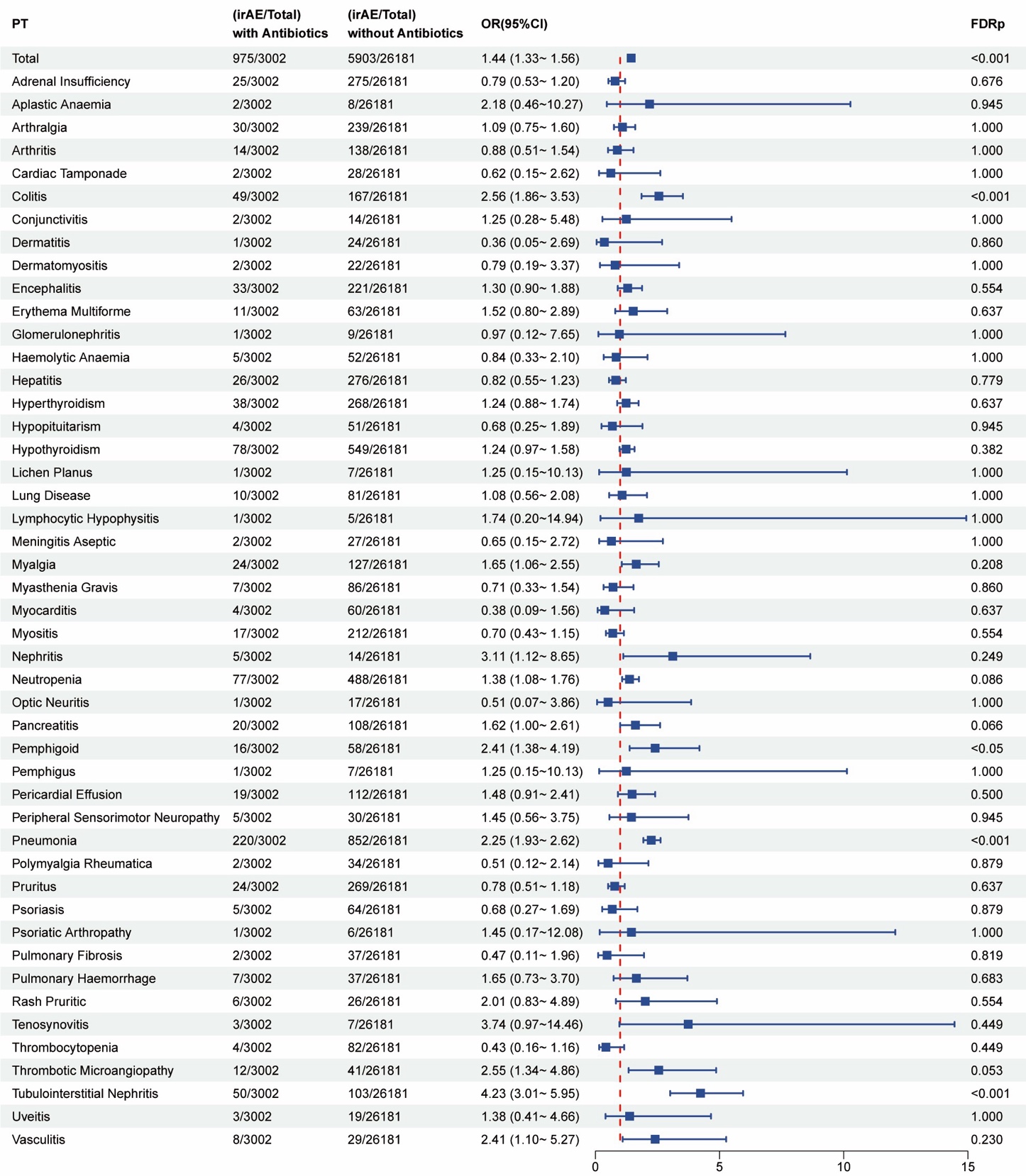


Figure S4：Forest plot illustrating the association between antibiotic use and various irAEs in patients treated with PD-L1 inhibitors.


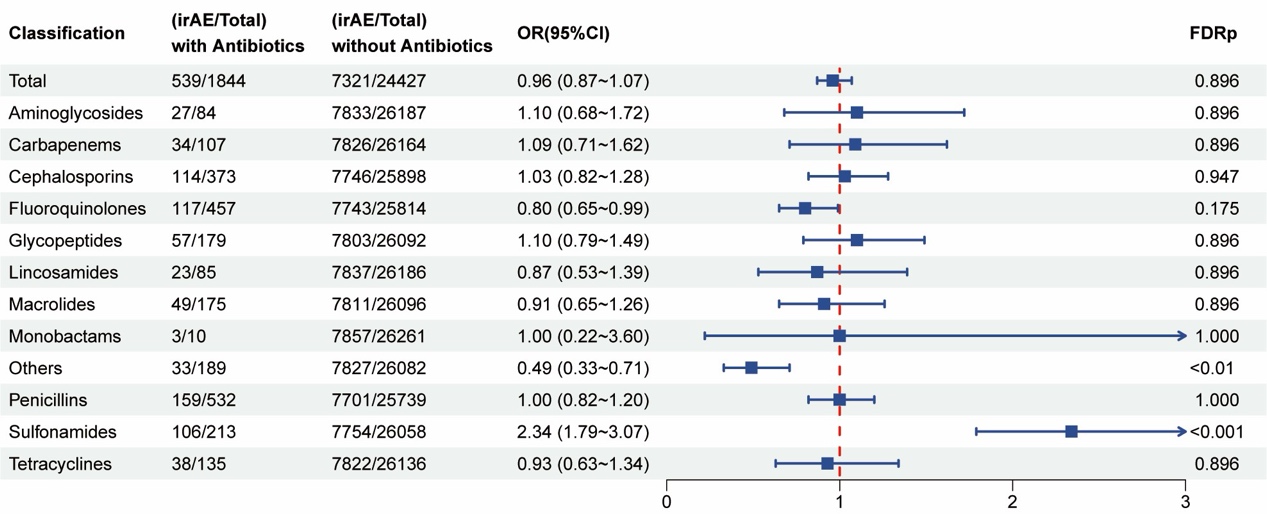


Figure S5. Multivariable logistic regression analysis of irAE risk associated with CTLA-4 inhibitor treatment combined with different classes of antibiotics.

Figure S6.Box plots and histograms showing the distribution of reported time to first irAE among patients receiving PD-L1 inhibitor therapy, comparing antibiotic co-reporting versus non-co-reporting group
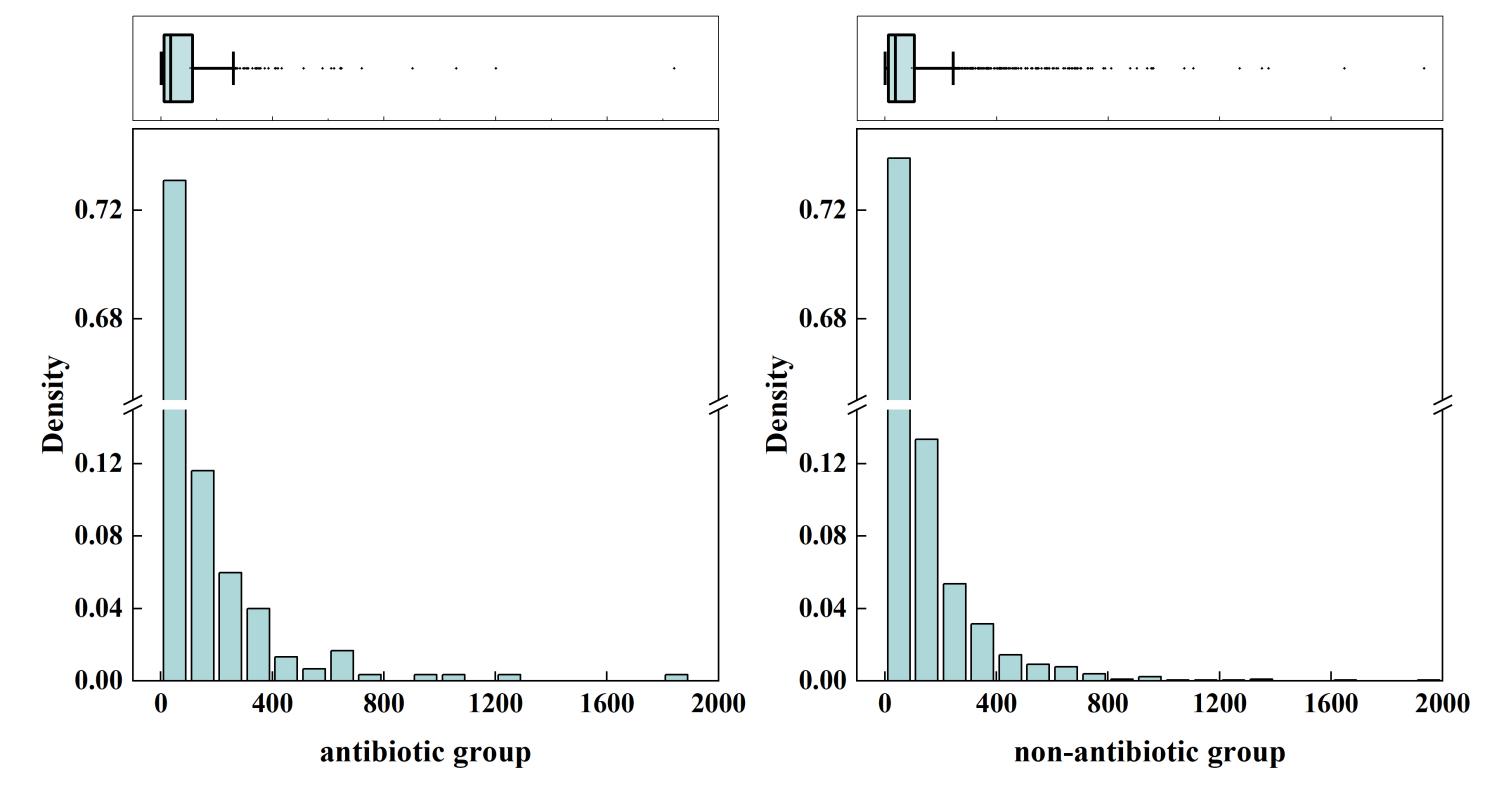
s.

Figure S7. Box plots and histograms showing the distribution of reported time to first irAE among patients receiving CTLA-4 inhibitor therapy, comparing the antibiotic co-reporting group versus the non-co-reporting group.


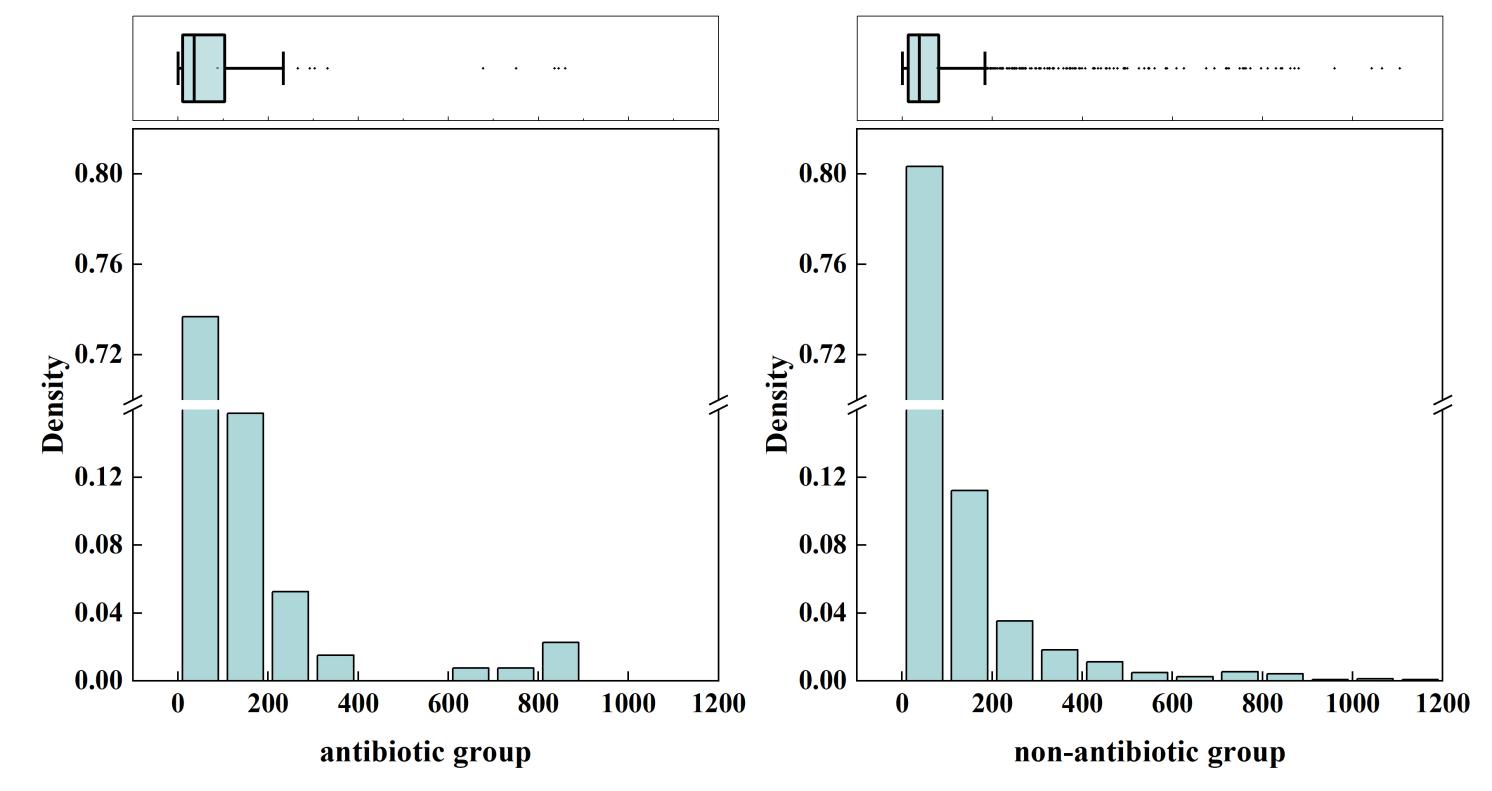


**
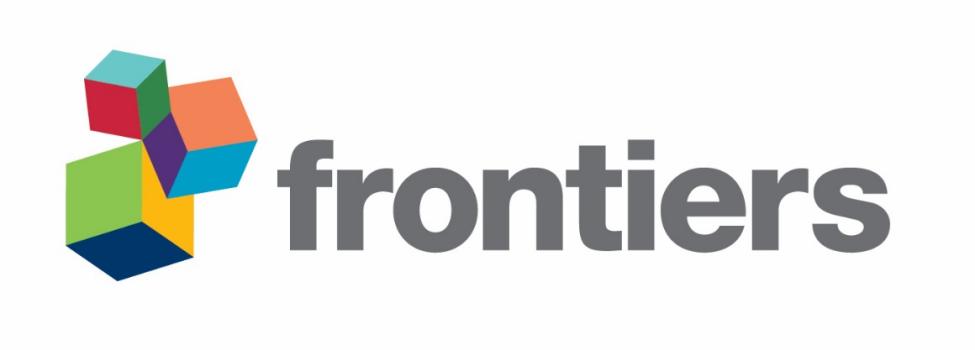
**
